# Menstrual blood-derived mesenchymal stromal cells: impact of preconditioning on the cargo of extracellular vesicles as potential therapeutics

**DOI:** 10.1186/s13287-023-03413-5

**Published:** 2023-07-28

**Authors:** María Ángeles de Pedro, Esther López, Francisco Manuel González-Nuño, María Pulido, Verónica Álvarez, Ana María Marchena, Christian Preußer, Witold Szymański, Elke Pogge von Strandmann, Johannes Graumann, Francisco Miguel Sánchez-Margallo, Javier G. Casado, María Gómez-Serrano

**Affiliations:** 1 Stem Cell Therapy Unit, Jesús Usón Minimally Invasive Surgery Centre, 10071 Cáceres, Spain; 2 RICORS-TERAV Network, ISCIII, 28029 Madrid, Spain; 3 Institute for Tumor Immunology, Center for Tumor Biology and Immunology, Philipps University, 35043 Marburg, Germany; 4 Core Facility Extracellular Vesicles, Center for Tumor Biology and Immunology, Philipps University, 35043 Marburg, Germany; 5 Institute of Translational Proteomics, Biochemical/Pharmacological Center, Philipps University, 35043 Marburg, Germany; 6 Immunology Unit, University of Extremadura, 10003 Cáceres, Spain; 7 Institute of Molecular Pathology Biomarkers, University of Extremadura, 10003 Cáceres, Spain

**Keywords:** Extracellular vesicles (EVs), Exosomes, High-throughput proteomics, Menstrual blood, Mesenchymal stromal cells (MSCs), Microvesicles, Preconditioning

## Abstract

**Background:**

Mesenchymal stromal cells (MSCs) have been shown to exert their therapeutic effects through the secretion of broad spectrum of paracrine factors, including extracellular vesicles (EVs). Accordingly, EVs are being pursued as a promising alternative to cell-based therapies. Menstrual blood-derived stromal cells (MenSCs) are a type of MSC that, due to their immunomodulatory and regenerative properties, have emerged as an innovative source. Additionally, new strategies of cell priming may potentially alter the concentration and cargo of released EVs, leading to modification of their biological properties. In this study, we aimed to characterize the EVs released by MenSCs and compare their therapeutic potential under three different preconditioning conditions (proinflammatory stimuli, physioxia, and acute hypoxia).

**Methods:**

MenSCs were isolated from five healthy women. Following culturing to 80% confluence, MenSCs were exposed to different priming conditions: basal (21% O_2_), proinflammatory stimuli (IFNγ and TNFα, 21% O_2_), physioxia (1–2% O_2_), and acute hypoxia (< 1% O_2_) for 48–72 h. Conditioned media from MenSCs was collected after 48 h and EVs were isolated by a combination of ultra-filtration and differential centrifugation. An extensive characterization ranging from nano-flow cytometry (nFC) to quantitative high-throughput shotgun proteomics was performed. Bioinformatics analyses were used to derive hypotheses on their biological properties.

**Results:**

No differences in the morphology, size, or number of EVs released were detected between priming conditions. The proteome analysis associated with basal MenSC-EVs prominently revealed their immunomodulatory and regenerative capabilities. Furthermore, quantitative proteomic analysis of differentially produced MenSC-EVs provided sufficient evidence for the utility of the differential preconditioning in purpose-tailoring EVs for their therapeutic application: proinflammatory priming enhanced the anti-inflammatory, regenerative and immunomodulatory capacity in the innate response of EVs, physioxia priming also improves tissue regeneration, angiogenesis and their immunomodulatory capacity targeting on the adaptive response, while acute hypoxia priming, increased hemostasis and apoptotic processes regulation in MenSC-EVs, also by stimulating immunomodulation mainly through the adaptive response.

**Conclusions:**

Priming of MenSCs under proinflammatory and hypoxic conditions affected the cargo proteome of EVs released, resulting in different therapeutic potential, and thus warrants experimental exploration with the aim to generate better-defined MSC-derived bioproducts.

**Supplementary Information:**

The online version contains supplementary material available at 10.1186/s13287-023-03413-5.

## Background

In the last decade, mesenchymal stromal cells (MSCs) have become the dominant cellular source in the field of stem cell therapy, most notably those isolated from bone marrow, adipose tissue, and umbilical cord [1]. Their therapeutic action has been investigated in multiple preclinical and clinical trials for the treatment of a plethora of disorders, including cardiovascular, neurodegenerative, and immune diseases, among others [2]. Despite the beneficial therapeutic properties demonstrated in vitro, clinical trials based on MSC therapy have been plagued by inconsistent outcomes with moderate to poor efficacy [1, 2].

Considering that the effect of MSCs depends on the tissue of origin, new cell sources are being explored to evaluate their clinical applicability. The endometrium, which undergoes monthly cycles of regeneration, differentiation, and detachment throughout a woman's reproductive period, has been highlighted as a potential source of stromal cells with broad regenerative properties [3]. The use of menstrual blood-derived stromal cells (MenSCs) has gained increasing attention since their discovery in 2007 [4]. As compared to other sources, these cells stand out by their noninvasive and ethically unproblematic procurement, high proliferation rate, short doubling time, chromosomal stability, and low immunogenicity, among other advantages [5]. Unlike other MSCs, MenSCs can stably expand for at least 20 passages [6], maintaining their morphology and showing no symptoms of senescence until P10 [7].

A wealth of evidence points to the therapeutic effect of MSCs being strongly dependent on their paracrine activity. This fact has led numerous studies to explore the therapeutic potential of the MSC secretome as a whole, and more specifically extracellular vesicles (EVs) derived from them. MSC-derived secretome and EVs have emerged as alternatives to cell therapy [8], which has been shown to display numerous side effects including host cell rejection, detrimental effects in the pulmonary microvasculature, as well as potential tumor development [9]. Indeed, secretome- rather than cell-based therapy was shown to overcome many of those in addition to facilitated handling and application [9].

Among the many advantages of cell-free therapy over cell therapy are: (1) easy storage; (2) feasibility for safety, dosage, and activity evaluation; (3) reduced immunogenicity; (4) absence of genotoxicity associated with long-term cell cultures; (5) ease of administration [8], in addition to other manufacturing advantages (reduced costs, production scaling, and lower regulatory burden) [10, 11].

The cellular secretome may be divided into two major components: a soluble fraction, composed of growth factors, cytokines, and other metabolites, and a vesicular fraction to a large extent represented by EVs [10]. EVs are nanoparticles delimited by a lipid bilayer with a diameter of 50–1000 nm and contain a molecular cargo including proteins, mRNAs, and miRNAs [12] as well as lipids and other metabolites [13]. Based on their route of biogenesis, EVs may be further subdivided into three different types: microvesicles, exosomes, and apoptotic bodies [14], although classification remains fluid given differences in biogenesis, size, content, and function, as well as an exceedingly active EV research community [15]. Evidence suggests that MSC-derived EVs are able to partially mimic the biological effects of the parental cells, rendering them an alternative to cell-based therapies [14, 16]. Thus, the characterization of their molecular cargo is essential to understand the biological function and its potential therapeutic application. For MenSC-derived EVs, the miRNA content in the secretome has been previously studied by our research group [17]. In contrast, a comprehensive characterization of the MenSC- EV associated proteome remains elusive.

The modulation of biological properties of MSC using various preconditioning strategies is well established in the field and represents a valuable tool to enhance their therapeutic effects as well as products derived from them [2]. Thus, preconditioning MenSCs using different culture conditions is expected to modulate EV concentration and cargo. Two of the most conventional preconditioning strategies are the exposure to inflammatory stimuli and/or hypoxic treatment: proinflammatory priming has been mainly explored with the goal to modulate inflammation and stimulate angiogenesis in injured tissues [2], whereas hypoxia has been applied to enhance regenerative potential by upregulating genes involved in processes such as inflammation, migration, proliferation, or angiogenesis [8, 18].

Therefore, the present work set out to achieve two objectives: (1) to define the EV-associated proteome produced by MenSCs; and (2), to determine how proinflammatory and hypoxia priming modify it. To the best of our knowledge, this work provides for the first time a quantitative proteomic characterization of primed MenSCs-derived EVs, a central step towards the understanding and the optimization of their pharmacological potential.

## Methods

### Culture and characterization of MenSCs

MenSCs were obtained from blood collected in a menstrual cup from five healthy premenopausal women (under 40 years) with regular cycles and without any type of hormonal treatment. All donors gave written informed consent to participate in the study. The experimental procedures were approved by the Ethics Committee of the Jesús Usón Minimally Invasive Surgery Center. MenSCs were isolated and expanded as previously described [19]. Briefly, cells were cultured in Dulbecco's modified Eagle's medium (DMEM) supplemented with 10% fetal bovine serum (FBS) (Gibco, Thermo Fisher Scientific, Bremen, Germany), 1% penicillin/streptomycin, and 1% glutamine (Thermo Fisher Scientific) at 37 °C and 5% CO_2_. The medium was changed every 2–3 days. The experiments here described were performed in passes P4-P8. In addition, phenotypic analysis of MenSCs was performed by flow cytometry, using FACSCalibur™ cytometer (BD Biosciences, CA, USA) equipped with CellQuest software (BD Biosciences). Following the guidelines of the International Society for Cellular Therapy (ISCT) [20], MenSCs were characterized using the following panel of surface markers: CD11B, CD14, CD29, CD31, CD34, CD44, CD45, CD73, CD90, CD105, CD117, CD146, HLA-I, HLAII, STRO1, and SUSD2 (Additional file 4: Table S1).

### Preconditioning conditions

To assess a possible enhancement of the MenSC therapeutic potential, cells were exposed to three different types of preconditioning: proinflammatory (PI, *n* = 5) and two hypoxic conditions, physioxia (PHY, *n* = 5) and acute hypoxia (AH, *n* = 5). In the PI priming condition, MenSCs, at 80% confluence and passages P4–P8, were cultured with IFNγ and TNFα (100 ng/ml, Miltenyi Biotec Inc, Auburn, CA, USA) for 72 h, while in the hypoxic priming conditions (PHY and AH), cells were exposed to 1–2% and < 1% oxygen concentration respectively for 48 h. Control cells were cultured in parallel in basal or normoxic conditions at 21% O_2_ (B, *n* = 5).

### Conditioned media collection and EV isolation

After preconditioning, cells were washed with PBS three times and cultured in serum-free DMEM without phenol red supplemented with 1% penicillin/streptomycin and 1% insulin–transferrin–selenium (ITS, Thermo Fisher Scientific) for 48 h. After this time, the conditioned medium was collected, centrifuged at 1000×*g* for 10 min at 4 °C, 5000×*g* for 20 min at 4 °C to remove dead cells and debris, and, subsequently filtered through 0.45- and 0.22-µm filters. After these steps, the secretome fraction was concentrated by centrifugation at 4000×*g* for 40 min at 4 °C, using a 3 kDa MWCO Amicon® Ultra device (Merck-Millipore, MA, USA). For EV isolation, the concentrated secretome was ultracentrifuged at 110,000×*g* for 2 h in a MAX-XP ultracentrifuge equipped with a TLA-45 fixed-angle rotor (Beckman Coulter, Krefeld, Germany), and the resulting pellet was washed with 0.1 µm filtered PBS (1 ml) and centrifuged again with the same settings. Finally, the supernatant was removed, and isolated EVs were suspended in 50–100 µl of filtered PBS and stored at − 80 °C for further analysis. When samples from different donors were pooled, concentrated secretome samples (AMICON samples) were equally mixed based on protein concentration (*n* = 5 donors) and EVs were isolated by ultracentrifugation as described.

### EV characterization

#### Electron microscopy

The morphological characteristics of EVs were confirmed by transmission electron microscopy (TEM). An equally mixed pool of EVs (*n* = 5 donors) was analyzed as previously described [21]. Briefly, 10 µl of EV suspension was fixed in an equal volume of 4% paraformaldehyde, and a small volume was transferred on a formvar-carbon-coated EM grid. After 20 min, air-dried grids were washed with PBS and incubated for 5 min in 1% glutaraldehyde. Afterwards, the grids were washed and transferred to a uranyl-oxalate solution, followed by a 10 min incubation on ice in a 4% uranyl acetate and 2% methylcellulose mixture in a 1/9 ratio, respectively. Excess fluid was removed and the grids were air-dried for 5–10 min. EVs were imaged with a Zeiss EM 900 at 80 kV, fitted with a 2 k slow-scan CCD camera (TRS).

#### Nanoflow cytometry (nFC)

Nanoflow cytometry (nFC) was applied for measuring particle concentration and size of EVs using a NanoAnalyzer equipped with a 488 nm laser and two single photon-counting avalanche photodiodes (ADP) (NanoFCM, Inc., Nottingham, UK) with calibration settings used at the EV Core Facility Marburg. Briefly, a monodisperse silica nanoparticles cocktail (68–155 nm, Cat. S16M-Exo, NanoFCM, Inc.) and 200 nm polystyrene beads (QC Beads, Cat. S08210, NanoFCM, Inc.) with a defined concentration of 2.08 × 10^8^ particles/mL were used for size and concentration calibration, respectively, at a sampling pressure of 1 kPa. Background subtraction was based on 0.1 µm filtered PBS solution. Dilution of EV samples was individually adapted to achieve a total number of analyzed particles between 2500 and 12,000 events in 1 min according to the manufacturer´s instructions. All samples were analyzed using the NF Profession V2.0 software (NanoFCM, Inc.).

Detection of tetraspanin-positive EVs was also performed by nFC. Briefly, EV pooled samples (10^9^ particles per sample) were stained individually with 200 ng of FITC-coupled anti-human CD9 (Clone HI9a, Cat. 312104, Biolegend), PE-coupled anti-human CD63 (Clone H5C6, Cat. 353004, Biolegend) or CD81 (Clone 5A6, Cat. 349506, Biolegend) in a final volume of 100 µl filtered PBS, ensuring optimal detection performance [22]. Corresponding isotype controls (Biolegend) were used as negative controls. Antibodies were prepared in 0.1 µm filtered PBS in advance and centrifuged for 15 min at 10,000×*g* at 4 °C prior to applying to the EVs. EVs and antibodies were incubated for 1 h at 37 °C and 600 rpm in the dark, followed by washing by adding 1 ml of 0.1 µm filtered PBS and a 110,000×*g* centrifugation for 1 h and 40 min at 4 °C using the Optima™ MAX-XP ultracentrifuge and TLA-45 fixed-angle rotor (Beckmann Coulter). The supernatant was discarded and the EV pellet was suspended in 50 µL PBS. Dilution for nFCM acquisition within the optimal range of events (< 10,000 total particles) was tested individually. All samples were acquired at 1 kPa pressure for 1 min and % of fluorescence positive events (gated in FITC-A and PC5-A channels, respectively), and size distribution was calculated using the NF Profession V2 software (NanoFCM, Inc.).

#### Immunoblotting

EV samples were homogenized in RIPA (10X) solution by vortexing and incubation on ice for 15 min and a freeze and thaw cycle at -20 °C, followed by the addition of Laemmli buffer (5×, 255 mM Tris–HCl, 50% glycerin, 5% (w/v) SDS, 0.05% (w/v) bromophenol blue). Laemmli buffer was additionally supplemented with 250 mM DTT when reducing conditions for protein detection were desired. All samples were incubated for 5 min at 95 °C, thawed, and run on a 10% SDS protein gel and transferred to Hybond ECL nitrocellulose membranes according to standard procedures. Membranes were blocked for 1 h with 2.5% (w/v) non-fat milk powder in TBS buffer containing 0.2% Tween-20 (TBS-T) and probed O/N at 4 °C with primary antibody solutions (Additional file 4: Table S2). Blots were washed and incubated with either anti-mouse IgG-HRP-conjugated secondary antibody (Cat. 7076S, Cell Signalling, Leiden, Netherlands) or fluorescently labeled anti-rabbit IgG-IRDye 800CW (Cat. 926-32211, LI-COR Biosciences) for 1 h at 1:10,000 dilution in TBS-T. Both detection methods were combined. Chemiluminescent bands were detected by prior incubation with Immobilon Forte Western HRP substrate Millipore (Cat. WBLUF0500, Merck, Germany) and both methods were visualized using a ChemiDoc MP system (Bio-Rad Laboratories, Inc.). Blots were exposed at different acquisition times. Optical densities of the immunoreactive bands were measured using Image Lab analysis software version 5 (Bio-Rad Laboratories, Inc.).

### Proteomics analysis

#### Sample preparation and digestion

EVs were collected and isolated from individual samples preconditioned by different stimuli (B, PI, PHY, and AH, *n* = 5 per group). A total of 1.93 × 10^9^ ± 8.7 × 10^8^ particles were used on average (± SD) and each sample volume was adjusted to 50 μl of PBS. Samples were lysed by incubation with 50 μl of 8% sodium lauroyl sarcosinate (SLS) solution (final concentration of 4%) at 95 °C for 10 min, followed by reduction and alkylation through addition of DTT to a final concentration of 10 mM and incubation at 95 °C for 10 min, and iodoacetamide to a final concentration of 13 mM and incubation for 30 min at 25 °C, respectively. A modified version of the SP3 method [23] was used for further sample preparation on an in-house made magnetic rack. Protein binding was performed in a final concentration of 70% anhydrous acetonitrile solution at neutral pH with subsequent washes with 70% ethanol and 100% anhydrous acetonitrile (ACN). After acetonitrile removal, beads were resuspended in 50 μl of 50 mM TEAB buffer and 0.5 ug of trypsin (Promega, Madison, Wisconsin, USA) was added. Protein digestion was performed overnight, at 37 °C. Next, sample volumes were reduced to approximately 5 μl in a SpeedVac concentrator (Thermo Fisher Scientific). Peptide binding to beads was initialized by the addition of 100% ACN to its’ final concentration above 98%. Beads were washed twice using the same solvent. Peptides were eluted by the addition of 40 μl of 0.1% formic acid and transferred to MS vials. Peptide concentration was estimated using the fluorimetric Pierce Quantitative Peptide Assays and sample volumes were adjusted to achieve equal concentrations.

#### Mass spectrometry acquisition

Purified peptides were analyzed by liquid chromatography–tandem mass spectrometry (MS) carried out on a Bruker Daltonics timsTOF Pro instrument connected to a Bruker Daltonics nanoElute instrument. Approximately 300 ng of peptides were loaded onto a C18 precolumn (Thermo Trap Cartridge 5 mm, µ-Precolum TM Cartridge/PepMap TM C18, Thermo Scientific) and then eluted in the backflush mode with a gradient from 98% solvent A (0.15% formic acid) and 2% solvent B (99.85% acetonitrile and 0.15% formic acid) to 17% solvent B over 36 min, continued from 17 to 25% of solvent B for another 18, then from 25 to 35% of solvent B for another 6 min over a reverse-phase high-performance liquid chromatography (HPLC) separation column (Aurora Series Emitter Column with CSI fitting, C18, 1.6 μm, 75 μm × 25 cm, Ion Optics) with a flow rate of 400 nl/min. The outlet of the analytical column with a captive spray fitting was directly coupled to the MS instrument. Data were acquired using a data-independent acquisition (DIA) paradigm using a default method provided by Bruker. In short, spectra were acquired with a fixed resolution of 45,000 and mass range from 100 to 1700 m/z for the precursor ion spectra and a1/k0 range from 0.6 to 1.6 V s/cm2 with 100 ms ramp time for ion mobility, followed by DIA scans with 21 fixed DIA windows of 25 m/z width, ranging from 487.5 to 1012.5 m/z.

#### Spectra identification

Peptide spectrum matching and label-free quantitation were performed using DIA-NN [24] using library-free search against the Homo sapiens Uniprot database (20,397 Swiss-Prot entries, October 2022), parametrized as documented in the Additional file 1. In brief, output was filtered to a 1% false discovery rate on precursor level. Deep learning was used to generate an in silico spectral library for library-free search. Fragment m/z was set to a minimum of 200 and a maximum of 1800. In silico peptide generation allowed for N-terminal methionine excision, tryptic cleavage following K*,R*, a maximum of one missed cleavage, as well as a peptide length requirement of seven amino acid minimum and a maximum of 30. Cysteine carbamidomethylation was included as a fixed modification and methionine oxidation (maximum of two) as a variable modification. Precursor masses from 300 to 1800 m/z and charge states one to four were considered. DIA-NN was instructed to optimize mass accuracy separately for each acquisition analyzed and protein sample matrices were filtered using a run-specific protein q-value.

#### Downstream bioinformatic data processing

Data analysis and statistics were carried out on DIA-NN’s “report.tsv” using the R package autonomics (version 1.1.7.9) [25], including proteins with a protein *q* value < 0.01 and requiring detection of three or more precursors (Np) in at least three donors as additional filtering criteria. MaxLFQ [26] values were used for quantitation and missing values imputed. Differential expression analysis was evaluated by autonomics employed Bayesian moderated t-test as implemented by limma [27]. Differential abundant proteins (DAPs) were selected considering a cut-off of log_2_FC = 1 and an adjusted *p* value < 0.01 following to Benjamini-Hochberg (BH) correction for multiple hypothesis testing. Functional and pathway enrichment analyses were performed using DAVID (Database for Annotation, Visualization, and Integrated Discovery, https://david.ncifcrf.gov/) [28] using R, version 4.2.2. The top significantly enriched terms were plotted with R-studio using the ggplot2 package in score dotplots. The global functional characterization of basal MenSCs-EVs (B-EVs), represented as a network combining GO and pathways category, was performed using Metascape (https://metascape.org/). Additionally, to examine the priming effect on the EV cargo, all GO significantly enriched terms (cut-off Benjamini *p* value < 0.05) were further clustered and divided into biologically relevant groups with the pathfindR package [29]. The most representative categories, according to the lowest* p* value, from each cluster were visualized in a heatmap, using a score assigned to each protein term. This term-score was calculated with the MaxLFQ value of each DAP to subsequently infer whether a protein term was globally activated or repressed in each sample. Voronoi plots for the most representative pathways included in the immune system major term (R-HSA-168256) were visualized with the Reactome web tool (https://reactome.org/). Additional statistical differences between the different preconditioning stimuli against basal, control conditions were determined by one-way ANOVA test (Tukey´s post hoc correction).

## Results

### MenSCs and derived EVs characterization

**Fig. 1:**
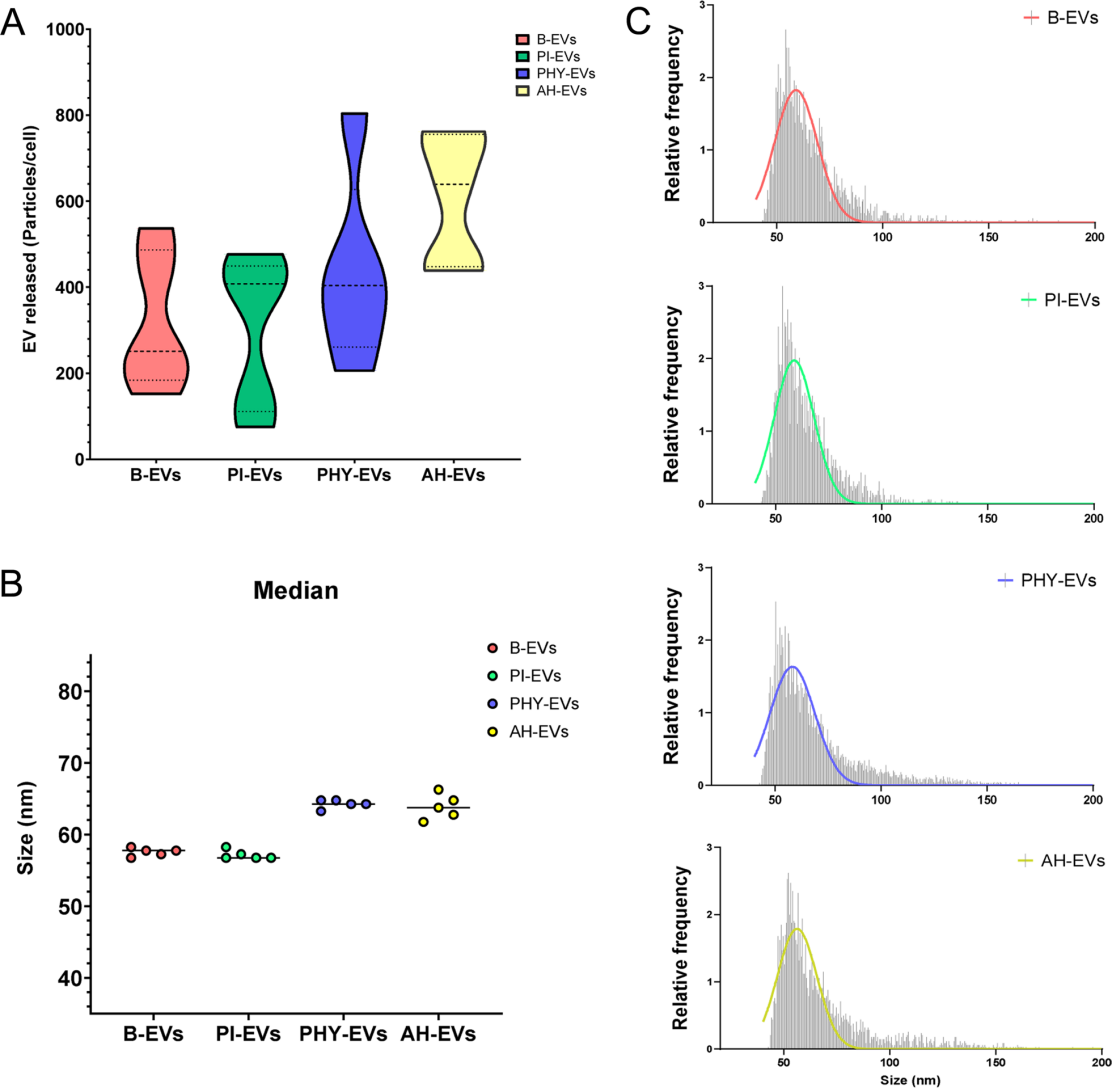
Characterization of MenSC-EV samples by nFC. MenSCs were exposed to different pre-conditioning conditions for 48–72 h. Subsequently, conditioned media from MenSCs were collected in DMEM serum-free media (1%ITS, 1% P/S) for 48 h and EVs were isolated and characterized by combining ultra-filtration and differential centrifugation methods (see details in “Methods” Section). **A** EV release by MenSCs exposed to different priming conditions. EV release was estimated based on the number of particles released per cell exposed to basal (B, red), pro-inflammatory stimuli (PI, green), physioxia (PHY, blue) or acute hypoxia (AH, yellow) pre-conditioning. Samples from five different donor cell lines were individually analyzed by nano flow-cytometry (nFC). **B** Median size particles (nm) analyzed in panel A. The line indicates averaged values for *n* = 5 donors. **C** Sizing profile of representative pooled samples. A pool of *n* = 5 donor conditioned media samples was mixed 1:1 and EVs subsequently isolated in order to decrease inter-individual variability. Histograms represent particles detected by nFC using a bin size of 0.5 nm (small EV range, 40–200 nm). Non-linear gaussian fit curves are also plotted in the interest of visualization. EVs, extracellular vesicles; MenSCs, menstrual blood-derived stromal cells; B-EVs, EVs released by basal MenSCs; PI-EVs, EVs released by pro-inflammatory primed MenSCs; PHY-EVs, EVs released by physioxia cultured MenSCs; AH-EVs, EVs released by acute hypoxia cultured MenSCs

**Fig. 2:**
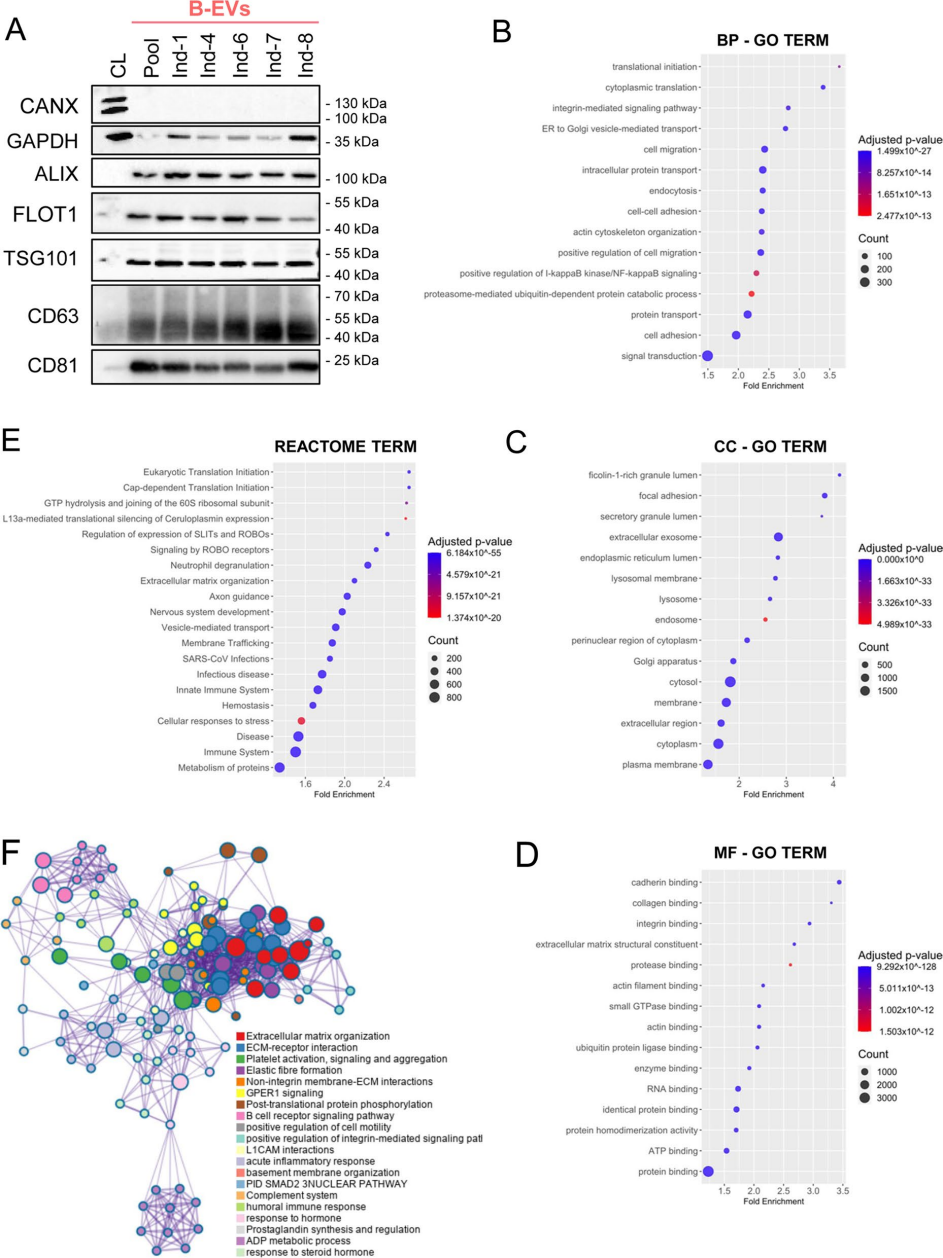
Profile of Basal MenSCs-derived EVs cargo proteome. **A** Characterization of the protein content of EV preparations by SDS-PAGE according to MISEV Guidelines [30]. Detection of proteins in category 1a (as tetraspanins CD63 and CD81), 2a (as TSG101, ALIX, and FLOT1), 2b (as GAPDH), and 4c (as CANX) are shown. A cell lysate, CL, (7.5 μg of total extract) was used as a control parallel to 4 × 10^9^ particles isolated from the equally 1:1 pooled or the corresponding individual EV samples (*n* = 5). Molecular weights are indicated. Uncropped blots are presented in Additional file 2 and Additional file 3. GO enrichment of proteins identified in B-EVs. The most significant terms were clustered by the three GO subontologies: **B** Biological process (BP); **C** cellular component (CC); **D** molecular function (MF). **E** Reactome enrichment chart. The most significant processes are highlighted in blue and the least significant processes in red according to Benjamini-Hochberg-adjusted *p* values. Larger dots in the graphs indicate a greater number of proteins involved. Only the top 20 categories are shown. **F** Network graph obtained from Metascape composed of significantly enriched categories colored according to the functional cluster they belong to. Node size depends on the number of proteins annotated within the corresponding category. EVs, extracellular vesicles; MenSCs, menstrual blood-derived stromal cells; B-EVs, EVs released by basal MenSCs

Flow cytometry showed that MenSCs were positive for CD29, CD44, CD73, CD90, CD105, CD117, CD146, HLA-I, SUSD2, and negative for CD11b, CD14, CD31, CD34, CD45, HLA-II, and STRO-1 (Additional file 4: Fig. s1). No significant differences were detected between the conditions analyzed. In addition to the phenotypic characteristic of MenSCs, we also characterized EV samples derived from them according to the minimal information for studies of extracellular vesicle (MISEV) guidelines [30] (Fig. 1). Results obtained by nFC indicated that EV release (estimated by the total number of particles isolated per cell) varied among donors but remained largely comparable for the basal and proinflammatory conditions. MenSC primed using hypoxic conditions tended to an enhanced rate of EV production (Fig. 1A). Secreted particles were confirmed to fall within the small EV range (40–200 nm), presenting a median size of 60–65 nm under all conditions and without apparent differences between donors within the same group (Fig. 1B). Size profiles of equally mixed pools (Fig. 1C) and electron microscopy analyses (Additional file 4: Fig. s2) corroborated these observations. Moreover, EV preparations from basal MenSCs were further evaluated using immunoblotting (Fig. 2A, Additional file 2, Additional file 3), targeting marker proteins in both pooled and individual samples in relation to their total cell lysate counterparts. Notably, tetraspanins such as CD63, CD81, and biogenesis factors like ALIX, TSG101, flotillin-1 (*FLOT1*), or GAPDH were present in all EV preparations. In contrast, calnexin (*CANX*) was not detectable in any of the samples.

Following EV characterization, we proceeded to the comparative quantitative shotgun proteomic analysis of their corresponding proteinaceous cargo. Spectra identification software (DIA-NN) identified 5350 protein groups. After dropping 99 potential contaminants, 41 without replication (within subgroup), 5210 protein groups were retained for further analysis. Furthermore, for this dataset, 422 protein groups have systematic missing values (NAs), 2024 protein groups have random NAs and 2764 protein groups have no NAs were imputed (*data not shown*). In a first step, these proteins detected in B-EV preparations were examined for their subcellular origin. For this purpose, protein terms identified with three or more peptide precursors (Np) in at least three samples were functionally annotated and a relative abundance estimation was performed based on the total Np sum. We focused on three major Gene Ontology (GO) categories: *lysosome* (GO:0005764) and *cytoskeleton* (GO:0005856) as read-outs for cross-contamination, and *exosomal* (GO:0070062) as a read-out of “purity for small EVs”, despite its limited number of members. Our analysis confirmed *exosome* (GO:0070062) as the most abundant annotation term, in contrast to potential co-isolated contaminants that only accounted for < 10% of the total protein identifications (Additional file 4: Fig. s3A). Additionally, the number of identified proteins in B-EVs (*n* = 4151) was compared to the number of proteins reported in the Vesiclepedia database (http://microvesicles.org/), a compendium of all proteins associated with EV studies [31]. As expected, most of the identified proteins in B-EVs overlapped with Vesiclepedia annotated proteins (89.7%) (Additional file 4: s3B), further confirming the reliability of this study and extending previous results.

### Proteome profile of basal MenSC-EVs underlines its immunomodulatory properties

To the best of our knowledge, the present study provides the deepest MenSC-EV proteome on record to date and thus provides the opportunity to further explore the biological functionality of B-EVs. To this end, GO enrichment analyses for the three ontology subcategories were carried out and presented in a score dotplot (Fig. 2B, C, and D). The *Biological Process* results showed that B-EV-associated proteins were mainly related to processes such as cellular transport, including vesicle-mediated transport or cell adhesion and migration (Fig. 2B). As expected, the terms identified in the *Cell Component* category (Fig. 2C) were related to extracellular organelles, membranes, and cytosolic parts, with the most significant category being the *extracellular exosome* (*p* value = 0). On the other hand, the *Molecular Function* category demonstrated a clear enrichment in binding proteins (Fig. 2D).

Additionally, the biological annotation was also queried using the Reactome database. From this ontology, *Immune System* (*p *value = 1.51 × 10^–48^) arose as one of the most enriched pathways (Fig. 2E). Finally, the 50 most abundant proteins in B-EVs in terms of MaxLFQ values were analyzed with Metascape (https://metascape.org). This analysis revealed an important functional role of these proteins in processes associated with the immune system and extracellular matrix organization (Fig. 2F).

### Preconditioning of MenSCs significantly modifies the EV-associated proteome, including the tetraspanin markers

**Fig. 3:**
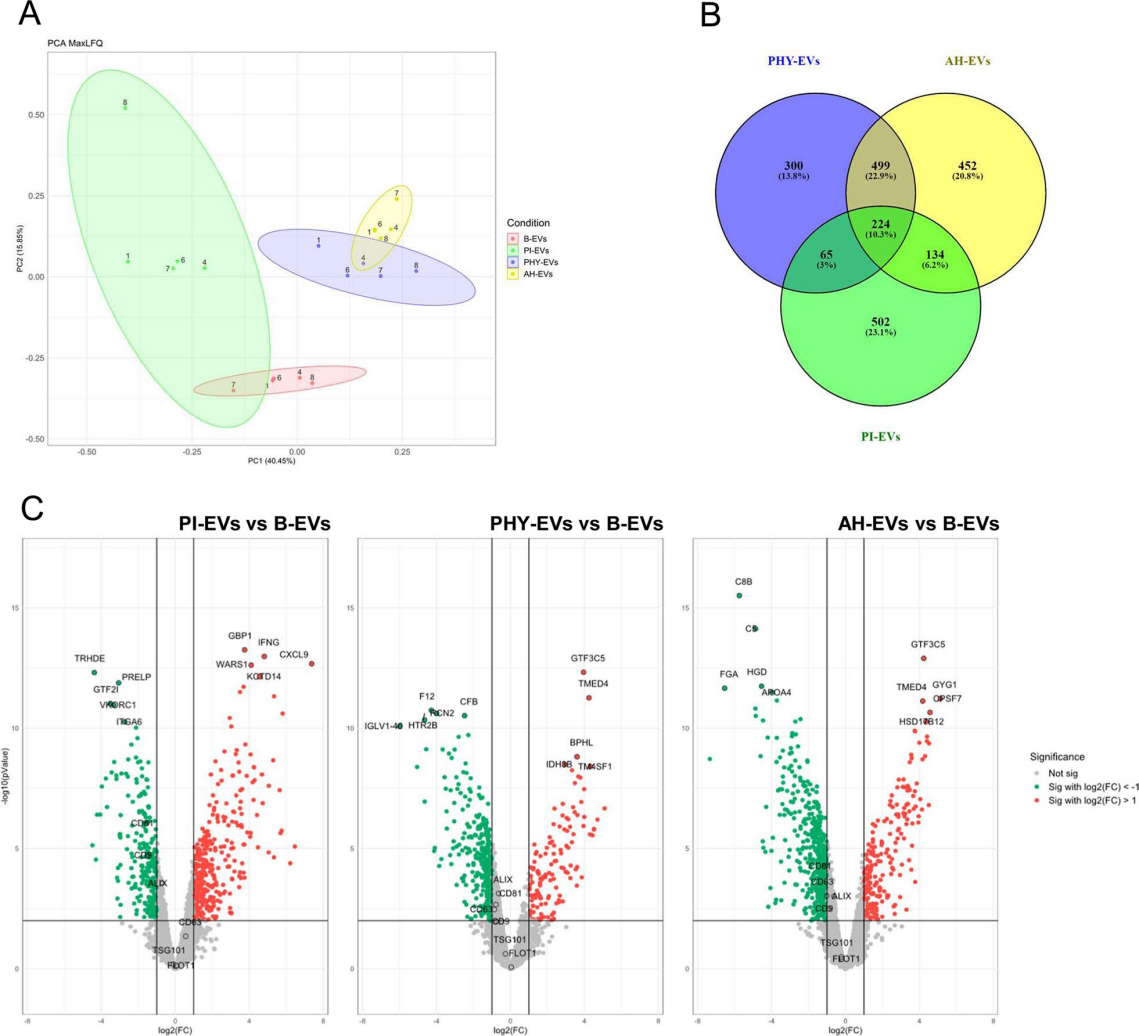
Proteomic alterations in EVs were obtained following different preconditioning of MenSCs. Proteomic data of different biogroups was filtered (detection in at least three donors) and comparatively analyzed. **A** Principal Component Analysis (PCA) showed a high level of clustering between biogroups. **B** Venn diagram depicting overlapping DAPs identified in the different EV samples. DAPs identified in B-EVs (red), PI-EVs (green), PHY-EVs (blue), and AH-EVs (yellow) are represented. **C** Volcano plots of differentially expressed proteins in PI-EVs (left), PHY-EVs (middle), and AH-EVs (right) vs. B-EVs. Values indicate the log2FC (X-axis) and –log10adjusted *p* value (Y-axis). Significantly *(p* < 0.01) increased (red dots, log2FC ≥ 1) and decreased (green dots, log2FC ≤ -1) proteins in the preconditioning *vs*. basal conditions are highlighted. Top-10 dysregulated proteins are depicted on the volcano plots, together with common EV markers. DAPs: differential abundant proteins; EVs, extracellular vesicles; MenSCs, menstrual blood-derived stromal cells; B-EVs, EVs released by basal MenSCs; PI-EVs, EVs released by pro-inflammatory primed MenSCs; PHY-EVs, EVs released by physioxia cultured MenSCs; AH-EVs, EVs released by acute hypoxia cultured MenSCs

Protein abundance differences between subsets of MenSC-EVs obtained under different preconditioning stimuli were analyzed. Principal component analysis (PCA) already showed appreciable clustering among the four biogroups (Fig. 3A). Statistical testing was then used to evaluate differences of EV proteomes from each priming condition against the basal group. Selecting DAPs using a fold change cut-off of log2FC = 1 and an adjusted *p* value < 0.01, show the protein distribution between biogroups using Venn diagrams (Fig. 3B). A total of 224 (10.3%) of the differential proteins were common to all MenSC-EV samples regardless of the priming conditions and 723 DAPs (33.2%) were shared between hypoxic pre-conditionings (Fig. 3B). Volcano plots show the distribution of protein abundance (log2FC scale) between datasets indicating an increase (red) or decrease (green) for each condition in comparison to B-EVs, with the top-10 most significant DAPs indicated (Fig. 3C). Key EV-associated markers are also indicated, independently of their significance in abundance changes (Fig. 3C).

**Fig. 4:**
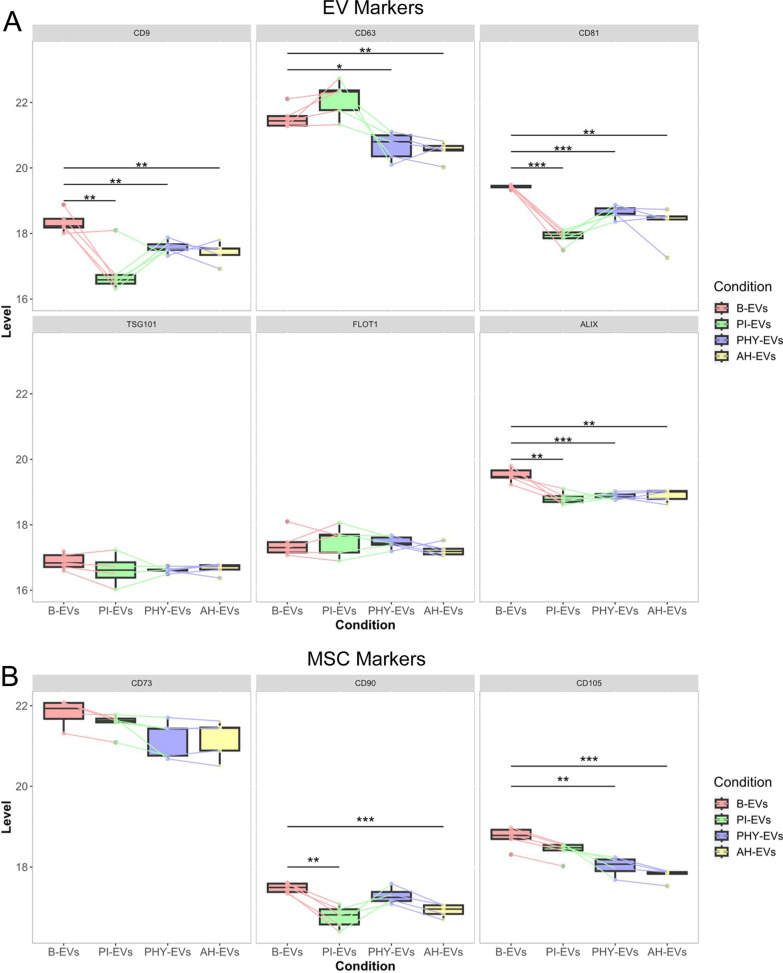
Changes in tetraspanins, EV biogenesis, and MSC markers in EV samples upon different preconditioning of MenSCs. **A** Abundance levels of the tetraspanins CD9, CD63, and CD81 (upper panel) and other EV biogenesis molecules like TSG101, FLOT1, and ALIX (lower panel) were individually evaluated among different EV groups. **B** The most representative MSC markers were also analyzed. Box plots indicate median protein abundance level based on MaxLFQ values in B-EVs (red), PI-EVs (green), PHY-EVs (blue), and AH-EVs (yellow) samples. Significant differences were tested by ANOVA one-way (Tukey´s post hoc test *vs*. basal conditions). *, *p* < 0.05; **, *p* < 0.005; and ***, *p* < 0.0005. EVs, extracellular vesicles; MenSCs, menstrual blood-derived stromal cells; B-EVs, EVs released by basal MenSCs; PI-EVs, EVs released by pro-inflammatory primed MenSCs; PHY-EVs, EVs released by physioxia cultured MenSCs; AH-EVs, EVs released by acute hypoxia cultured MenSCs

Interestingly, a deeper look into EV-associated proteins revealed that among all tetraspanins evaluated, CD63 was the most abundant followed by CD81 and CD9, irrespective of the preconditioning (Fig. 4A). An analysis of pooled EV samples by fluorescent nFC confirmed that EV-subpopulations CD63^+^, CD81^+^, CD9^+^ mimic this pattern only upon basal and proinflammatory priming, in contrast to hypoxic conditions in which the relative distribution of these subpopulations was clearly altered (Additional file 4: s4A). Differences at the proteomic level pointed to proinflammatory priming significantly decreasing CD9 and CD81 abundances on EVs, with no significant changes on CD63 but an apparent slight increase (Fig. 4A). Changes in CD63 followed the same variation pattern in terms of protein abundance and corresponding EV subpopulation frequency (Additional file 4: s4A). Both hypoxic preconditioning types, to the contrary, lead to a significant decrease in the three tetraspanin abundances (Fig. 4A), but a relative increase in CD9+ and CD81+ subpopulations (Additional file 4: Fig. s4A). The sizing profile of these CD9+, CD63+, and CD81+ subpopulations did not reveal substantial differences between them or basal EVs (Additional file 4: Fig. s4B). Moreover, the proteomics data further confirmed the presence of TSG101, FLOT1, and ALIX associated with all EVs. Whereas TSG101 and FLOT1 were stable between different preconditioning conditions, ALIX showed significant downregulation irrespective of the method (Fig. 4A). Taking together these observations, we propose TSG101 or FLOT1 as potential normalization markers for MenSC-EV studies, in contrast to any of the canonical tetraspanin markers, which showed significant differences at both proteomic level and EV-subpopulation distribution (Fig. 4A, Additional file 4: s4A).

Moreover, given the recent description of MSC markers on EVs [32], these molecules were given special consideration in our dataset. CD73, CD105, and CD90 were abundantly detected in MenSC-EVs (Fig. 4B), with CD73 protein as the most abundant one. It is noteworthy that CD73 did not show significant differences following any of the preconditioning conditions tested. However, the detection of CD90 was significantly diminished in PI and AH conditions as well as the abundance of CD105 in EVs obtained after both hypoxic exposures, even though MenSCs did not show significant dysregulation of any of these markers upon preconditioning at the cellular level (Additional file 4: Fig. s1).

### Proinflammatory or hypoxic preconditioning alters MenSC-EV biological properties

**Fig. 5:**
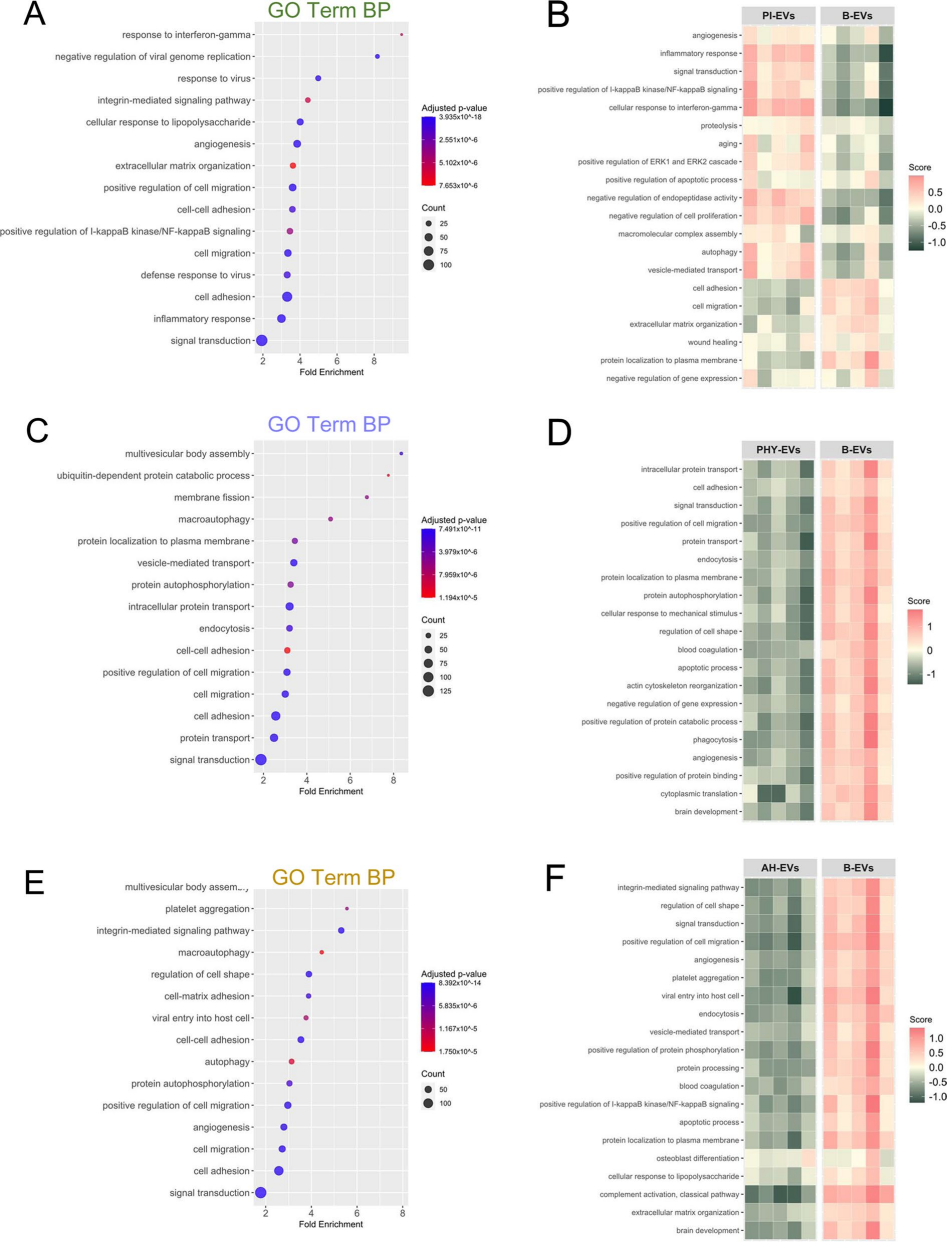
Comprehensive analysis of the MenSC-EV associated proteome according to biological function (GO ontology). A functional enrichment analysis was performed with the different DAP datasets to determine the *Biological Function* (BP) in which proteins were involved using DAVID. Dot-plots represent enriched GO BP terms among DAPs in PI-EVs **(A)**, PHY-EVs **(C)**, and AH-EVs **(E)** vs. B-EVs comparisons. A color-scale bar represents the level of significance for Benjamini-adjusted *p* values (blue, highest; red, least). The size of the dots indicates the number of proteins involved in each process. Clustering of the BP was carried out and represented in heatmaps with the most representative enriched terms from each cluster for the PI-EVs **(B)**, PHY-EVs **(D)**, and AH-EVs **(F)** vs. B-EVs comparisons. A protein score was calculated considering all proteins annotated within each category (see further details in Methods Section). The color scale of this score indicates a general up- (red) or down-regulation (green) in the preconditioning *vs*. basal conditions of these proteins. DAPs, differentially abundant proteins; EVs, extracellular vesicles; MenSCs, menstrual blood-derived stromal cells; B-EVs, EVs released by basal MenSCs; PI-EVs, EVs released by pro-inflammatory primed MenSCs; PHY-EVs, EVs released by physioxia cultured MenSCs; AH-EVs, EVs released by acute hypoxia cultured MenSCs

The biological function of DAPs identified under different priming conditions was explored by bioinformatic analyses using the GO database. In a first pass analysis, up- and down-regulated DAPs of each comparison were analyzed jointly. Here, enrichment analysis focusing on the *Biological Process* (BP) category revealed that inflammatory response, angiogenesis, as well as cell adhesion and migration processes were modified by proinflammatory preconditioning (PI) (Fig. 5A). Subsequently, the set of GO categories significantly enriched was clustered using the pathfindR package and the most representative GO category for each cluster (lowest *p* value) represented in a heatmap. Global GO activation or repression was inferred from the relative abundance values for the associated DAPs. This analysis found proteins involved in both the *inflammatory response* and *angiogenesis* up-regulated in PI-EVs, whereas proteins involved in processes of *wound healing*, *cell adhesion*, and *cell migration* were globally down-regulated in PI-EVs, as compared to B-EVs (Fig. 5B). In the case of physioxia priming (PHY), major enriched processes were related to *intracellular transport* and *cell adhesion* (Fig. 5C). Subsequent analysis including the abundance levels of DAPs involved in these GO categories showed a repression of all representative processes in PHY-EVs compared to B-EVs, which included processes such as *angiogenesis*, *apoptotic processes*, and *blood coagulation* (Fig. 5D). Lastly, for the condition of acute hypoxia priming (AH), pathways such as *cell adhesion*, *cell migration* and *angiogenesis* were enriched in DAPs (Fig. 5E), and similarly to the effect of PHY, all biological processes representative of the resulting clustering were repressed, including *angiogenesis*, *apoptosis*, *complement activation* but also *vesicle-mediated transport* (Fig. 5F).

### Preconditioning of MenSCs affects their immunomodulatory capacity through their EV-associated proteome alteration

**Fig. 6:**
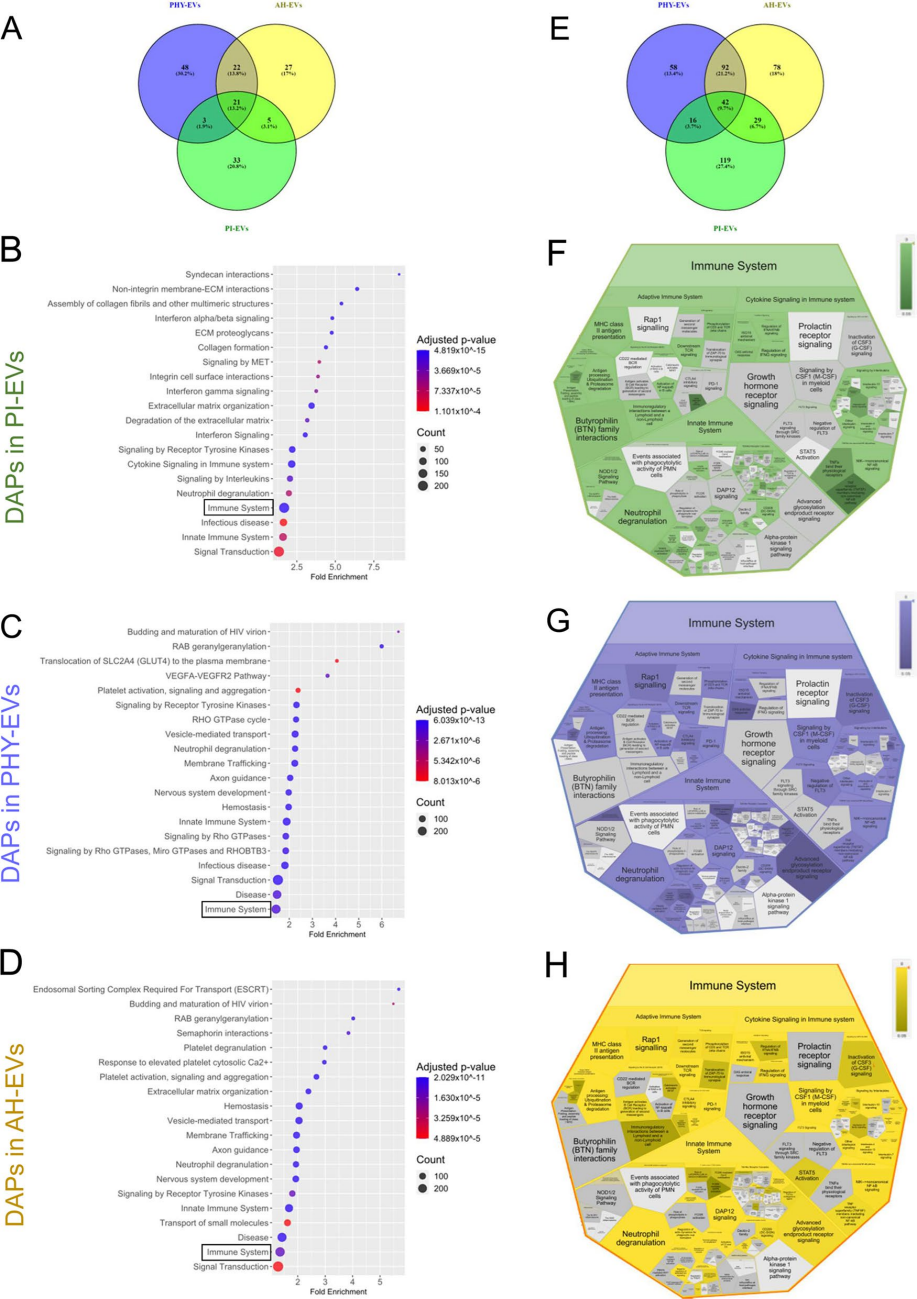
Reactome functional analysis. A pathway enrichment analysis was performed with the DAPs in each priming condition to determine over-represented pathways, using Reactome. **A** Venn diagram of the common significantly enriched pathways observed between priming conditions. Dot-plots with over-represented Reactome pathways among DAPs in PI-EVs **(B)**, PHY-EVs **(C),** and AH-EVs **(D)** vs. B-EVs. **(E)** Venn diagram of DAPs among different priming conditions annotated in common immune system pathways. Voronoi diagram of the over-represented pathways within the Immune System category (R-HSA:168,256) including the DAPs from PI-EVs, green **(F)**; PHY-EVs, purple **(G)**; AH-EVs, yellow **(H)** vs. B-EVs comparisons. Dot-plots representing the top-20 significant biological processes. A color-scale bar represents the level of significance for Benjamini-adjusted *p* values (blue, highest; red, least). The size of the dots indicates the number of proteins involved in each process. In the voronoi diagram, a color-scale indicates the statistical significance, the lighter the more significant. Gray color is used for pathways not represented. DAPs, differentially abundant proteins; EVs, extracellular vesicles; MenSCs, menstrual blood-derived stromal cells; B-EVs, EVs released by basal MenSCs; PI-EVs, EVs released by pro-inflammatory primed MenSCs; PHY-EVs, EVs released by physioxia cultured MenSCs; AH-EVs, EVs released by acute hypoxia cultured MenSCs

Based on the associated GO biological processes thus elucidated, the bioinformatics tool Reactome was used to analyze the DAPs and identify processes in which they were involved. Principal over-represented biological pathways (*p* value < 0.05) for each preconditioning approach compared to identify common and unique enriched pathways. Interestingly, only 21 differential pathways were found to be common among the biogroups (13.2%) and the majority of pathways appear to be characteristic of each priming condition (Fig. 6A). Our results further showed that after pro-inflammatory stimuli, EV-associated proteins were enriched in immune system pathways, such as *cytokine signaling in immune system*, *interferon signaling*, or *signaling by interleukins*, in addition to extracellular matrix organization pathways (Fig. 6B). On the other hand, after physioxia priming, proteins present in EV preparations were representative of vesicle transport and also immune system pathways. In this case, however, the latter was dominated by the *innate immune system* and *neutrophil degranulation* (Fig. 6C). Similarly, acute hypoxia exposure altered the vesicle transport and immune system protein component of EVs, in addition to the innate *immune system* and *neutrophil degranulation* pathways already mentioned and with platelet-associated pathways standing out as an additional category (Fig. 6D).

Considering that immune system pathways were prominently over-represented in all priming conditions; we investigate in more detail the potential differential effects of different priming conditions in these in particular. To this end, DAPs annotated for *Immune System* pathway (R-HSA-168256) were compared. A total of 42 immune system-related DAPs were found to be common between datasets (9.7%), with 119 PI-specific, 78 PHY-specific, and 78 HA-specific EV-associated DAPs (Fig. 6E).

Since most DAPs were priming condition-specific, we were encouraged to further investigate how priming affects minor pathways of the immune system super category. For this purpose, all-related *Immune System* (R-HSA-168256) pathways, were visualized per priming condition (Fig. 6F–H). In the *Innate Immune System* subdivision, *neutrophil degranulation* pathway was overrepresented for all priming conditions. *Complement cascade* was also shared by all biogroups, albeit significantly stronger enriched following AH priming. In contrast, *DAP12 signaling* was only enriched following hypoxic priming conditions. In the adaptive immune response, *PD-1 signaling* and *inhibitory CTLA4 signaling* were specific to hypoxia pre-conditioning in general but not PI priming. In contrast, the *immunoregulatory interactions between a lymphoid and a non-lymphoid cell* pathway were exclusive to PI. Regarding cytokine signaling, interleukin, 10, 4, and 13 pathways were shared by all conditions. However, *interleukin 20 and interleukin 7 signalin*g were only relevant following PI and AH priming, respectively. *CSF1* and *CSF3 signaling* were found to be specific following hypoxic preconditioning.

## Discussion

Although stem cell-based therapies using MSCs have long been considered a promising therapeutic option, their translation to the clinic has shown limited success and cell-free therapies are emerging as an alternative [33]. In contrast to other more commonly used MSC sources, MenSCs and -derived EVs are relative newcomers [34], although MenSC-derived small EVs were reported a few years ago by Lopez-Verrilli et al. [35]. Based on our previous findings studying the secretome collected from MenSCs [17, 36], here we aimed to characterize and compare the EVs released in the context of four different preconditioning conditions (basal, pro-inflammatory, physioxia, and acute hypoxia). To our knowledge, this is the first study that performs a comprehensive description of EVs released by MenSCs integrating state-of-the-art EV characterization techniques (including, but not limited to nano-flow cytometry) and high-throughput proteomics approaches.

Our results revealed no differences in terms of morphology, size, and number of EVs released between conditions, consistent with findings from other authors who have analyzed EVs released from different sources of MSCs [14, 37, 38]. However, our data showed a tendency to obtain more concentrated EV preparations of larger particle diameters under hypoxic conditions. This observation is in line with a significantly higher release of EVs upon hypoxic stimuli reported by Lo Sicco et al. [39]. EVs isolated from MenSCs met the minimum requirements laid out by the MISEV guidelines [30], including being positive for the tetraspanins and other EV markers. Interestingly, we also detected MSC markers on these preparations including CD73, CD90, and CD105, following the ISCT minimal criteria for MSC research. Altogether our data demonstrated the quality of our preparations [40], explicitly including conformity with applicable community guidelines. Notably, our preparations were obtained from MenSCs cultured no longer than P8, therefore avoiding confounding senescent effects [6]**.**

We next focused on defining the biological properties of EVs released by MenSCs under standard culture conditions. For this purpose, the potential implications of EV-associated proteins from MenSCs in different biological processes and signaling pathways were evaluated. Our analyses identified the presence of proteins involved in the modulation of both innate and adaptive responses and cell homing, reflecting the potent immunomodulatory capacity of B-EVs. These results not only highlighted the therapeutic potential of MenSC-EVs themselves but also agree with previously described proteomic signatures of MSC-EVs in different models [41]. Corroborative evidence was also found for the likely contribution of EVs to the immunomodulatory effect of the MenSC-secretome, as previously described in in vitro functional assays and in vivo observed in severe COVID-19 patients [17, 42]. In silico modelling of our proteomics data in a KEGG diagram pin-pointed some of the signaling mechanisms occurring in the alveolar cells leading to these therapeutic effects (Additional file 4: Fig. s5).

It is widely known that the composition of the MSC secretome, including the EV cargo, can be regulated by preconditioning strategies during in vitro culture. Preconditioning that mimics the harsh environment present at a site of injury, characterized, for instance, by heavy inflammation and low oxygen supply, may prime the cells to trigger the release factors enhancing the therapeutic properties of the secretome [2, 8, 37]. Our previous findings indicated that IFN-γ and TNF-α priming of MenSCs is an effective strategy to obtain a product with altered therapeutic potential [17, 43]. Mechanistically teasing apart the differences as reflected in the modulation of MenSC-EV protein cargo under different conditions, are key to success in their therapeutic application. As expected, our enrichment analysis on EV proteomes prominently revealed proteins involved in immunomodulatory processes, inflammatory response, angiogenesis, and adhesion. In line with these results, it has been shown that bone-marrow-derived MSCs stimulated with IFN-γ and TNF-α release EVs with increased capacity to interact with immune cells, potentially due to the enrichment of adhesion molecules in their EVs [44]. Moreover, an increase in proteins associated with chemotaxis and angiogenesis was identified in the EV-cargo released by pro-inflammatory primed umbilical cord-derived MSCs [38]. In in vivo experiments in an inflammatory bowel disease model animal, EVs from primed IFN-γ and TNF-α MSCs were further able to induce M2 macrophage polarization and modulate T cells activation and the expression of cytokines in the colon [45].

Mirroring the physiological niche of MenSCs may be another strategy to improve their EV therapeutic potential. The physiological oxygen concentration in the human uterus is 2% [46] and several studies have shown that EVs from MSC under hypoxic preconditioning harbor higher regenerative capacity as compared to those obtained under normoxia [8, 39]. Our results may partially explain such findings, as we found physioxia priming to alter molecular transport pathways, cell adhesion and migration processes, angiogenesis, and, as expected, the immune system-related proteins in the proteinaceous EV cargo. Interestingly, proteins involved in the angiogenesis category were mostly down-regulated in relation to B-EVs, but VEGFA, the most representative protein within this category, was up-regulated. Consequently, the pathway analyses also showed an overrepresentation of the VEGFA-VEGR2 pathway. In agreement with these findings, EVs from hypoxia-treated human adipose tissue-derived MSCs have been shown to display a high ability to increase angiogenesis mediated by the VEGF/VEGF-receptor [47]. As angiogenesis occurs during physiological and pathological processes [48], a therapeutic regulation of this mechanism by physioxia-stimulated MenSC-EVs may be an option. Further research is needed to determine to what extent the regenerative potential of PHY-EVs is applicable.

On the other hand, the pathophysiology of many diseases such as brain injury or myocardial infarction involves the presence of a hostile microenvironment with extremely low oxygen concentration. Exposing cell conditions mimicking this situation may stimulate the release of soluble factors in response to the pathological environment [18]. Therefore, we further evaluated the effect of preconditioning of MenSCs with acute hypoxia (AH). According to our results, those processes associated with cell migration and adhesion, immune response, and angiogenesis were altered in the EV-associated proteome by AH. These findings correlate with our recent in vitro studies using the secretome from AH-MenSCs [49].

Considering that EV-associated proteins involved in immune system pathways were modified by all priming conditions evaluated, a more detailed analysis of potentially altered mechanisms was carried out. Interestingly, this revealed that pathways related to the *Immune System* varied with the priming strategy. As expected, pathways involved in the regulation of inflammatory processes are prominent in EVs from PI priming, reflected in a proteomic profile with a significant increase in IFN-γ-related pathways. These signaling pathways are mainly involved in the coordination of host defense and immune surveillance, but also the establishment of adaptive immunity and the regulation of inflammation, apoptosis, and cell cycle [50, 51]. Focusing on their role in the regulation of inflammatory processes, it is interesting to note that some of the immunomodulatory drugs approved for autoimmune diseases act partially through the induction of IFN-γ pathways [52], so it may be relevant to assess the therapeutic effect of PI-EVs in autoimmune diseases such as rheumatoid arthritis (Additional file 4: Fig. s6). Moreover, IFNs play a major role in inflammasome activation [53], which is a key event in the inflammatory immune response, was found differentially enriched in PI conditions.

It is worth noting that preconditioning further differentially modulated interleukin signaling. In the case of PI-EVs, in particular, proteins involved in IL-1, IL-12, IL-10, and IL-20 signaling pathways were found to be enriched. IL-1 plays a key role in host defense as well as regulating stress and chronic inflammatory processes [54]. In addition, the IL-1 family is involved in the regulation of angiogenesis and vascular permeability [55]. IL-12 is another pro-inflammatory cytokine that acts as a major mediator between the innate and adaptive immune system, stimulating proliferation and cytotoxic activity of T lymphocytes and natural killer (NK) cells [56]. IL-10, on the other hand, is an anti-inflammatory cytokine with important immunoregulatory functions [57], which is also involved in remodeling stage of wound healing [58]. IL-20 belongs to the IL-10 cytokine family and has a critical role during the wound healing response [59]. Altogether, these findings highlight the potential of these EVs in tissue regeneration. The over-representation of all these pathways identifies PI-EVs as a promising therapeutic option to be evaluated in tissue regeneration strategies.

Moreover, our data indicate that PI priming favors the expression of MHC class I in EVs while hypoxia priming, does for class II. This may be indicative of fine-tuning T cytotoxic lymphocytes and T helper lymphocytes in the adaptative immune mechanism [60]. Hypoxia pre-conditioning was also found to stimulate GM-CSF signaling, which plays a critical role in the resolution of inflammation through macrophage function regulation [61]. This is in line with the earlier observation that hypoxic preconditioning is able to enhance macrophage polarization through M2 anti-inflammatory phenotypes [62] as a possible strategy in the resolution of inflammatory diseases [63]. In this regard, immune checkpoint inhibitors, such as PD-1 and CTLA-4, CD28 co-stimulation pathway, and TCR signaling were also highlighted in hypoxic MenSC-EVs corroborating their immunomodulatory effects and their effects on the maintenance of immune homeostasis [64]. Noteworthy, PD-1 and CTLA-4 are important cancer immunotherapy targets [65, 66] broadening the potential range of action of these EVs. Although a hypoxic environment during in vitro culture of MenSCs led to a release in EV-associated proteins related to common immune system pathways, slight differences in O_2_ pressure may have the potential to further modify and fine-tune their therapeutic effectiveness. Based on the finding that B cell receptor (BCR) signaling was only enriched in PHY-EVs, we additionally speculate that by adjusting hypoxic preconditioning, control of B cell immune responses through MenSC-EVs may be possible [67]. Similarly, proteins involved in the IL-7 signaling pathway were differentially stimulated with AH. This pathway contributes to host defense by regulating the development and homeostasis of immune cells, including T lymphocytes, B lymphocytes, and NK cells [68, 69].

## Conclusion

**Fig. 7:**
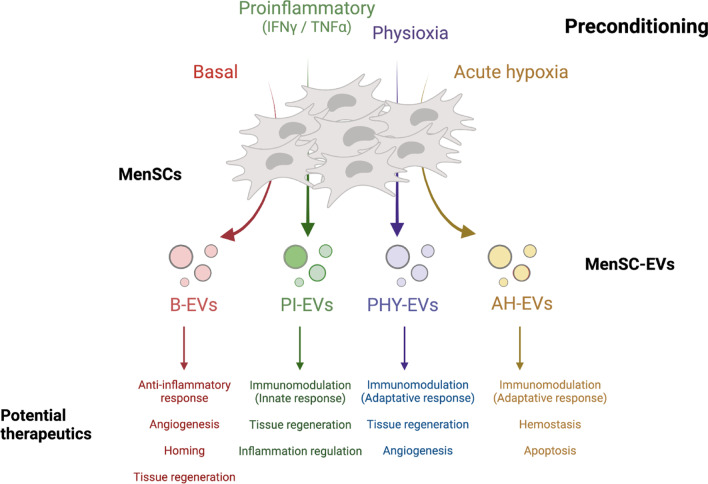
General overview of the main findings. EVs' therapeutic potential according to the pre-selected preconditioning conditions are depicted. EVs, extracellular vesicles; MenSCs, menstrual blood-derived stromal cells; B-EVs, EVs released by basal MenSCs; PI-EVs, EVs released by pro-inflammatory primed MenSCs; PHY-EVs, EVs released by physioxia cultured MenSCs; AH-EVs, EVs released by acute hypoxia cultured MenSCs. Created with BioRender.com

This study analyzes comprehensively for the first time the proteinaceous cargo of EVs released by human MSCs derived from menstrual blood undergoing different pre-conditioning regimes by using a high-throughput proteomic approach. We believe our findings provide useful information to pin-point priming strategies based on therapeutic goals (Fig. 7), and new original, sourced data to be modelled for the study of particular pathologies. Likewise, our comparative functional and pathway analyses based on the protein profile of MenSC-EVs contribute to a better understanding of the molecular mechanisms involved in the modulation of the immune response, among others, and offer essential insights for the future design of cell-free therapies.

The mass spectrometric raw data was deposited to the ProteomeXchange Consortium via the MassIVE partner repository (Dataset MSV000091644) and can be accessed using the data set identifier PXD043346. The rest of datasets used and/or analyzed in this manuscript are available on reasonable request to the corresponding authors.

## Supplementary Information

**Additional file 1**. Spectra identification parameters.

**Additional file 2**. Uncropped images in immunoblot analyses. Characterization of the protein content of EV preparations was performed by SDS-PAGE. Cell lysates, CL, (7.5 μg of total extract) were loaded and used as a control, in parallel to 4 × 109 particles isolated from the equally 1:1 pooled or the corresponding individual EV samples (*n* = 5). Extracellular vesicle (EVs) preparations were obtained from basal (B) and proinflammatory primed (PI) MenSCs. Only B-EVs samples were shown in Figure 2A. Blotting conditions are denoted (see further details in Supplementary Table 2). Molecular weights corresponding to the PageRuler Prestained Protein Ladder (Cat. 26616, Thermo Scientific) are indicated.

**Additional file 3**. Original TIFF images (600 dpi) of immunoblots. Fully uncropped blots shown in Figure 2A and Supplementary File 2 are included. Detection of CANX (**A**), CD63 (**B**), GADPH (**C**), and CD81 (**D**), which belong to the same SDS-PAGE performed under non-reducing conditions. Detection of ALIX (**E**), FLOT1 (**F**), and TSG101 (**G**), which belong to the same SDS-PAGE performed under reducing conditions. Bands shown in Figure 2A are indicated by red boxes.

**Additional file 4**. Supplementary Figures and Tables.

## Abbreviations

ACN: Acetonitrile
AH: Acute hypoxia priming
AH-EV: EVs released by acute hypoxia cultured MenSCs
BC: Biological process
B-EVs: EVs released by basal MenSCs
CC: Cellular component
DAPs: Differential abundant proteins
DIA: Data-independent acquisition
DMEM: Dulbecco’s modified Eagle’s medium
DTT: Dithiothreitol
EVs: Extracellular vesicles
FBS: Fetal bovine serum
FC: Fold change
GO: Gene ontology
HPLC: High-performance liquid chromatography
ISCT: International Society for Cell Therapy
ITS: Insulin–transferrin–selenium
MenSCs: Menstrual blood-derived stromal cells
MISEV: Minimal information for studies of extracellular vesicles
MF: Molecular function
MSCs: Mesenchymal stromal cells
MFI: Mean fluorescence intensity
MS: Mass spectrometry
NAs: Not available data
nFC: Nano-flow cytometry
O/N: Over-night
PCA: Principal component analysis
PI: Proinflammatory priming
PI-EVs: EVs released by pro-inflammatory primed MenSCs
PHY: Physioxia priming
PHY-EVs: EVs released by physioxia cultured MenSCs
TBS: Tris-buffered saline
TBS-T: 1× Tris-buffered saline, 0.1% tween® 20 detergent
TEAB: Triethyl ammonium bicarbonate buffer
TEM: Transmission electron microscopy
