# Supplementary material for: Menstrual blood-derived mesenchymal stromal cells: impact of preconditioning on the cargo of extracellular vesicles as potential therapeutics
Source: Stem Cell Res Ther. 2023 Jul 28;14:187. doi: 10.1186/s13287-023-03413-5 (PMC10386225; doi:10.1186/s13287-023-03413-5)
Supplement: Supplementary file 2 — Additional file 2. Uncropped images in immunoblot analyses. Characterization of the protein content of EV preparations was performed by SDS-PAGE. Cell lysates, CL, (7.5 μg of total extract) were loaded and used as a control, in parallel to 4 × 10^9^ particles isolated from the equally 1:1 pooled or the corresponding individual EV samples (n = 5). Extracellular vesicle (EVs) preparations were obtained from basal (B) and proinflammatory primed (PI) MenSCs. Only B-EVs samples were shown in Figure 2A. Blotting conditions are denoted (see further details in Supplementary Table 2). Molecular weights corresponding to the PageRuler Prestained Protein Ladder (Cat. 26616, Thermo Scientific) are indicated. [file 13287_2023_3413_MOESM2_ESM.pdf]

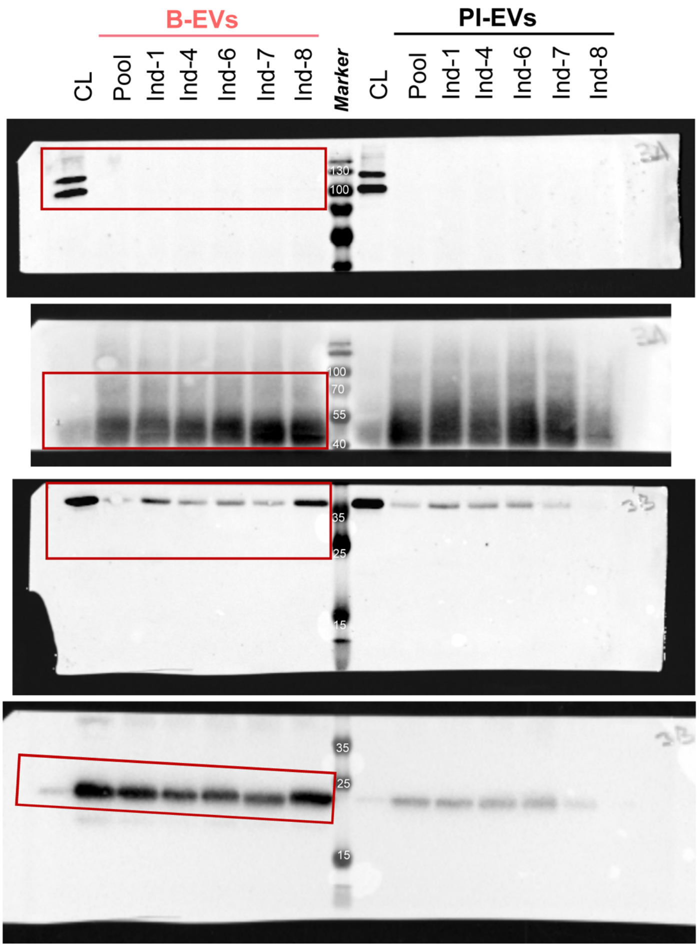


**CANX**
Non-reducing, gel 1 (blot 1)
(Detection by fluorescence, auto)

**CD63**
Non-reducing, gel 1 (blot 1)
(Detection by chemiluminescence, 1 sec)

**GAPDH**
Non-reducing, gel 1 (blot 2)
(Detection by fluorescence, auto)

**CD81**
Non-reducing, gel 1 (blot 2)
(Detection by chemiluminescence, 1 sec)

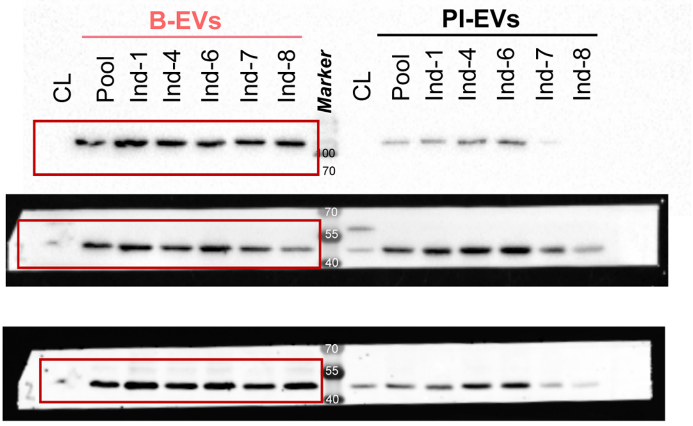


**ALIX**
Reducing, gel 2 (blot 1)
(Detection by chemiluminescence, 1 sec)

**FLOT1**
Reducing, gel 2 (blot 2)
(Detection by chemiluminescence, 4 sec)

**TSG101**
Reducing, gel 2 (blot 2)
(Detection by fluorescence, auto)

**Data shown in Figure 2A**
