# Supplementary figures and images for: Menstrual blood-derived mesenchymal stromal cells: impact of preconditioning on the cargo of extracellular vesicles as potential therapeutics
Source: Stem Cell Res Ther. 2023 Jul 28;14:187. doi: 10.1186/s13287-023-03413-5 (PMC10386225; doi:10.1186/s13287-023-03413-5)

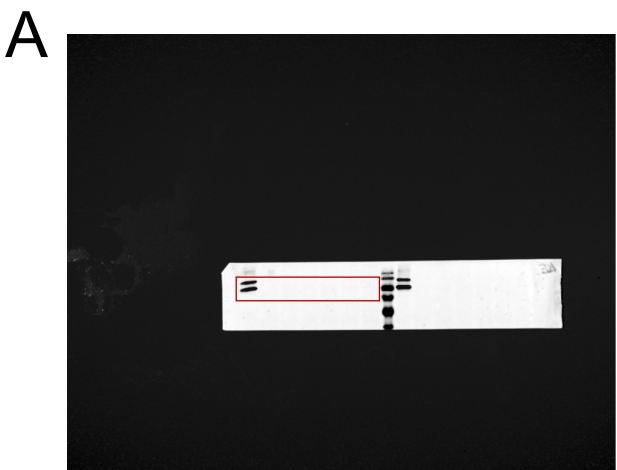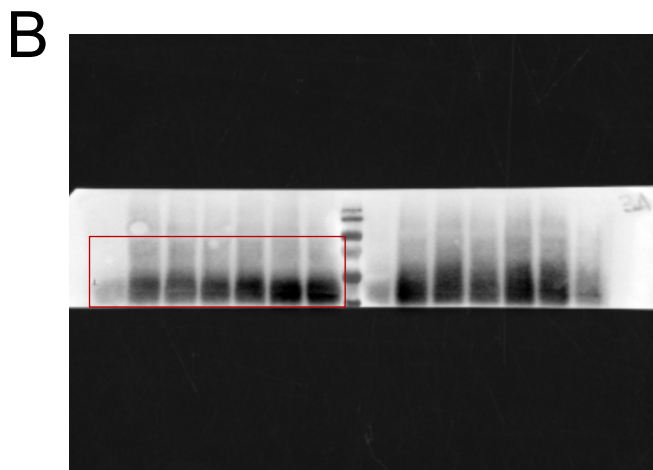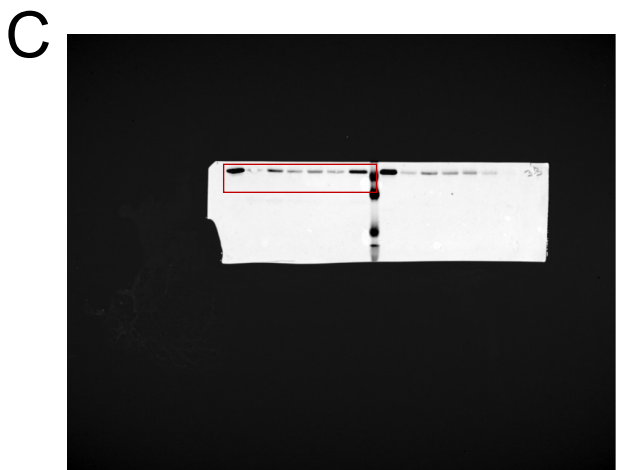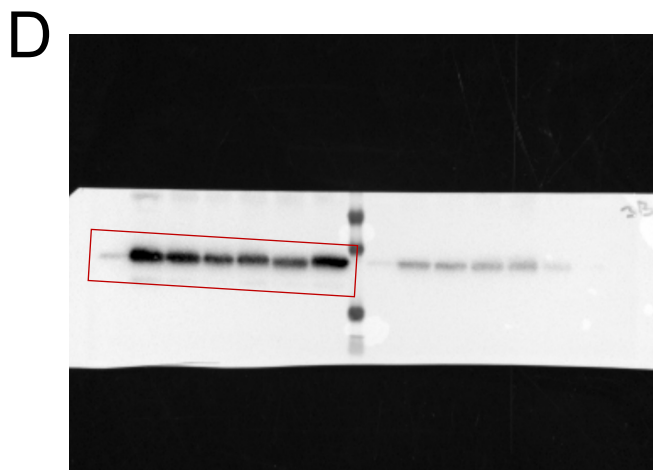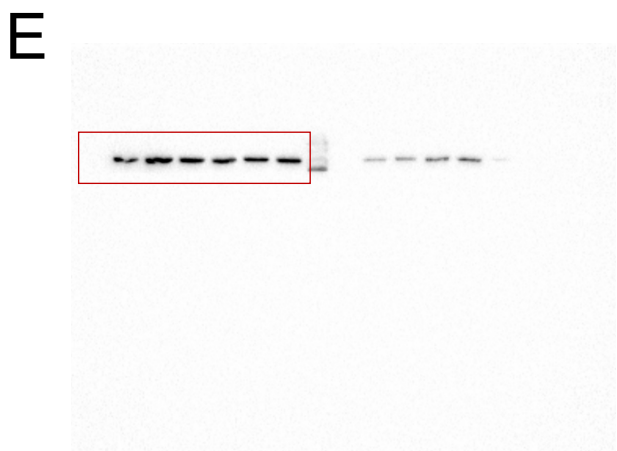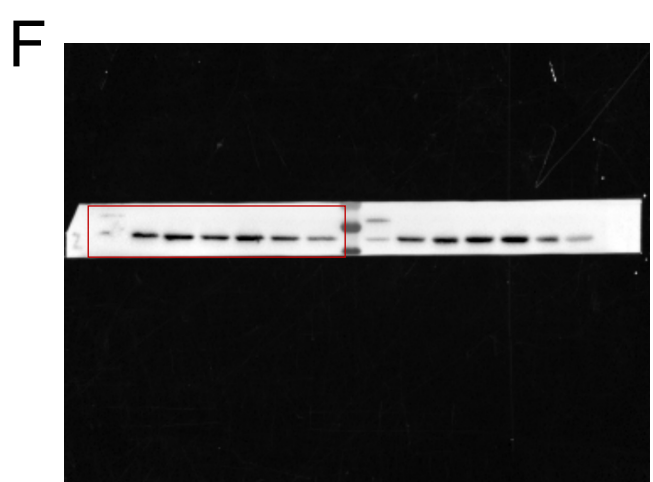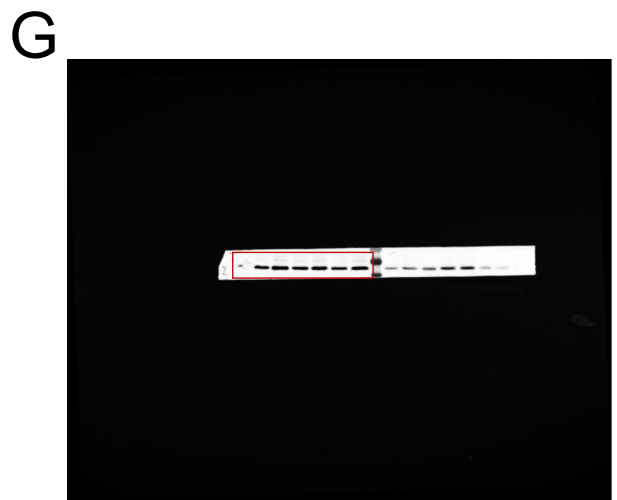

Supplement: Supplementary file 3 — Additional file 3. Original TIFF images (600 dpi) of immunoblots. Fully uncropped blots shown in Figure 2A and Supplementary File 2 are included. Detection of CANX (A), CD63 (B), GADPH (C), and CD81 (D), which belong to the same SDS-PAGE performed under non-reducing conditions. Detection of ALIX (E), FLOT1 (F), and TSG101 (G), which belong to the same SDS-PAGE performed under reducing conditions. Bands shown in Figure 2A are indicated by red boxes. [file 13287_2023_3413_MOESM3_ESM.pdf]
