# Supplementary material for: Menstrual blood-derived mesenchymal stromal cells: impact of preconditioning on the cargo of extracellular vesicles as potential therapeutics
Source: Stem Cell Res Ther. 2023 Jul 28;14:187. doi: 10.1186/s13287-023-03413-5 (PMC10386225; doi:10.1186/s13287-023-03413-5)
Supplement: Supplementary file 4 — Additional file 4. Supplementary Figures and Tables. [file 13287_2023_3413_MOESM4_ESM.docx]

**SUPPLEMENTARY INFORMATION**

**Menstrual blood-derived mesenchymal stromal cells: impact of preconditioning on the cargo of extracellular vesicles as potential therapeutics**

María Ángeles de Pedro^1,2^, Esther López^1,2*^, Francisco Manuel González-Nuño^1^, María Pulido^1^, Verónica Álvarez^1^, Ana M. Marchena^1,2^, Christian Preußer^3,4^, Witold Szymanski^5^, Elke Pogge von Strandmann^3,4^, Johannes Graumann^5^, Francisco Miguel Sánchez-Margallo^1,2^, Javier G. Casado^2,6,7^, and María Gómez-Serrano^3*^

1. Stem Cell Therapy Unit, Jesús Usón Minimally Invasive Surgery Centre, 10071 Cáceres, Spain
2. RICORS-TERAV Network, ISCIII, 28029, Madrid, Spain
3. Institute for Tumor Immunology, Center for Tumor Biology and Immunology, Philipps University, 35043 Marburg, Germany
4. Core Facility Extracellular Vesicles, Center for Tumor Biology and Immunology, Philipps University, 35043 Marburg, Germany
5. Institute of Translational Proteomics, Biochemical/Pharmacological Center, Philipps University, 35043 Marburg, Germany
6. Immunology Unit, University of Extremadura, 10003 Cáceres, Spain
7. Institute of Molecular Pathology Biomarkers, University of Extremadura, 10003 Cáceres, Spain

**Supplementary Figures**

**Supplementary Fig. 1. Analysis of MSC specific markers on differentially preconditioned MenSCs by flow cytometry.** Representative histograms of basal (red), pro-inflammatory primed (green), physioxia (blue) and acute hypoxia incubated (yellow) MenSCs were plotted for each of the analyzed surface markers. The opaque black histogram corresponds to the negative control. MenSCs: menstrual blood-derived stromal cells.


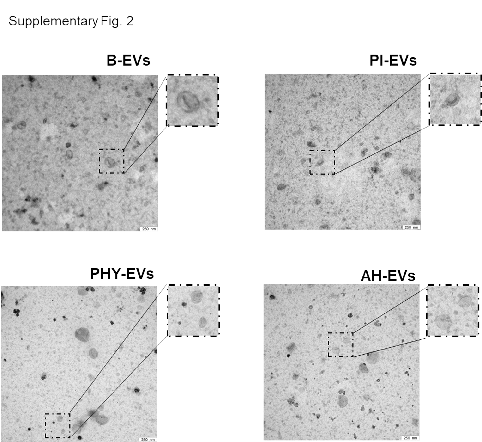


**Supplementary Fig. 2. Electron microscopy of MenSC-derived EV preparations.** Conditioned serum-free medium from different preconditioned MenSCs was collected for 48h and EVs were isolated by a combination of ultrafiltration and ultracentrifugation. Representative transmission electron microscopy pictures are shown. Scale bar, 250 nm. EVs, extracellular vesicles; MenSCs, menstrual blood-derived stromal cells; B-EVs, EVs released by basal MenSCs; PI-EVs, EVs released by pro-inflammatory primed MenSCs; PHY-EVs, EVs released by physioxia cultured MenSCs; AH-EVs, EVs released by acute hypoxia cultured MenSCs.

**Supplementary Fig. 3. Additional characterization of the basal MenSC-derived EVs proteome. (A)** Relative contribution of *exosomal* (GO:0070062), *lysosome* (GO:0005764) and *cytoskeleton* (GO:0005856) terms detected by proteomics. Basal EVs (B-EVs) were analyzed by LC/MS (further information in Methods section). For this analysis, the sum of detected precursors (Np) for the corresponding annotated proteins was normalized to the total Np detected for each individual sample, as an estimation of the relative abundance of these major categories. **(B)** Venn diagram showing the overlapping proteins identified in B-EVs samples (n = 4151) and the Vesiclepedia database (<http://microvesicles.org/>). MenSCs, menstrual blood-derived stromal cells; EVs, extracellular vesicles.

**Supplementary Fig. 4. Phenotypic analyses of MenSC-EVs by fluorescent nFC.** The phenotype of MenSC-EVs obtained upon different preconditioning conditions was analyzed using nFCM fluorescence analyses. **(A)** Staining results for CD9+ (black), CD63+ (grey) and CD81+ (purple) EV subpopulations released. Stacked bars indicate the cumulative frequency (% of total events analyzed) of single-stained subpopulations for each EV preconditioning type. **(B)** Sizing profile of CD9 (black), CD63 (grey) and CD81 (purple) positive events (within 40 – 200 nm range). Data represent the relative frequency of total gated, positive events with a bin size of 0.5 nm. A total of 10^9^ particles were used as equal staining input per marker and condition. Equally pooled samples (n = 5 donors) were analyzed in order to improve the homogeneity of the samples. All samples were analyzed in duplicates.


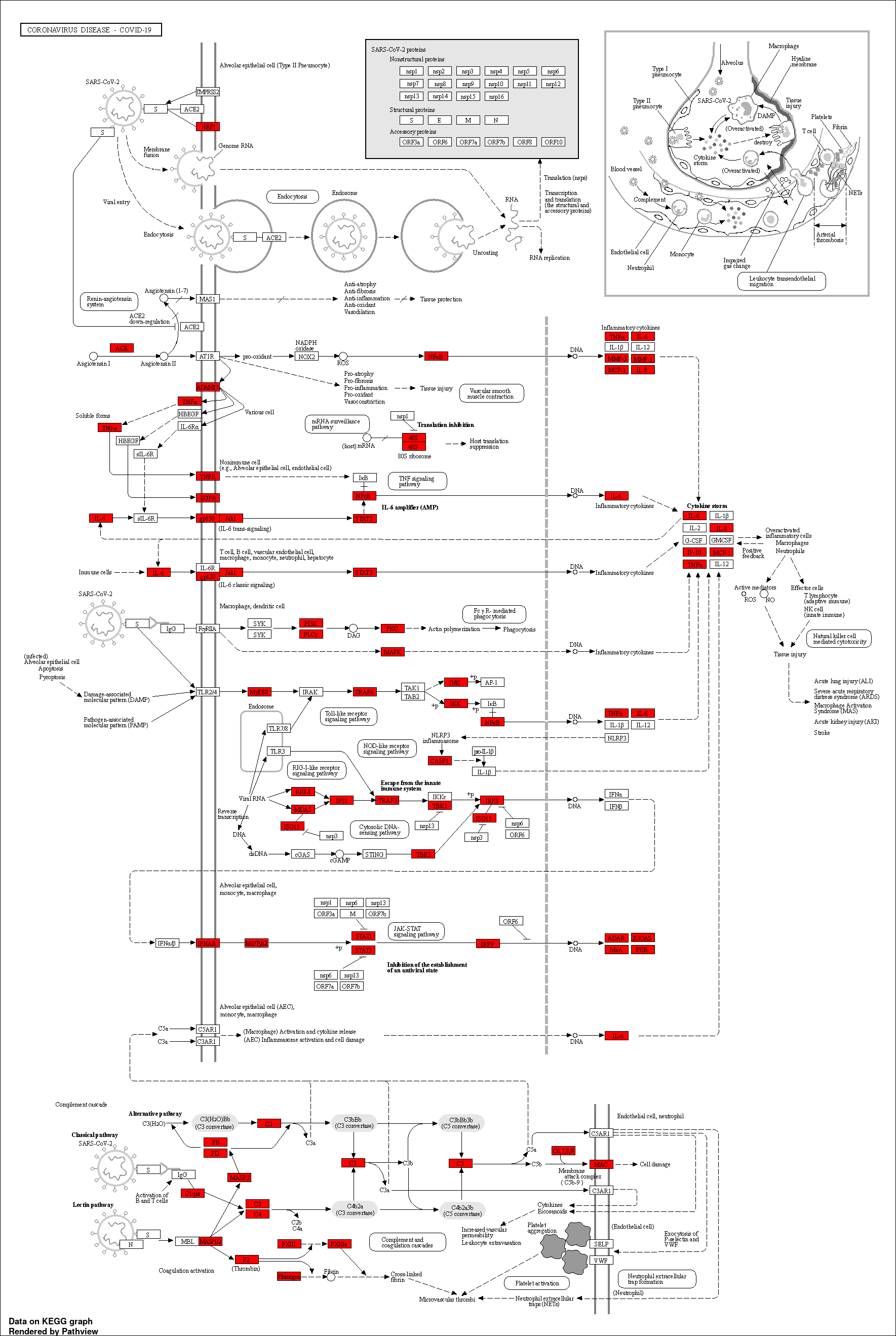


**Supplementary Figure 5. Therapeutical potential of B-EVs and underlying mechanisms in COVID-19.** Red nodes in the diagrams denote identified proteins in the corresponding EV-associated proteome. This KEGG diagram was created with ShinyGO 0.77 (http://bioinformatics.sdstate.edu/go/).


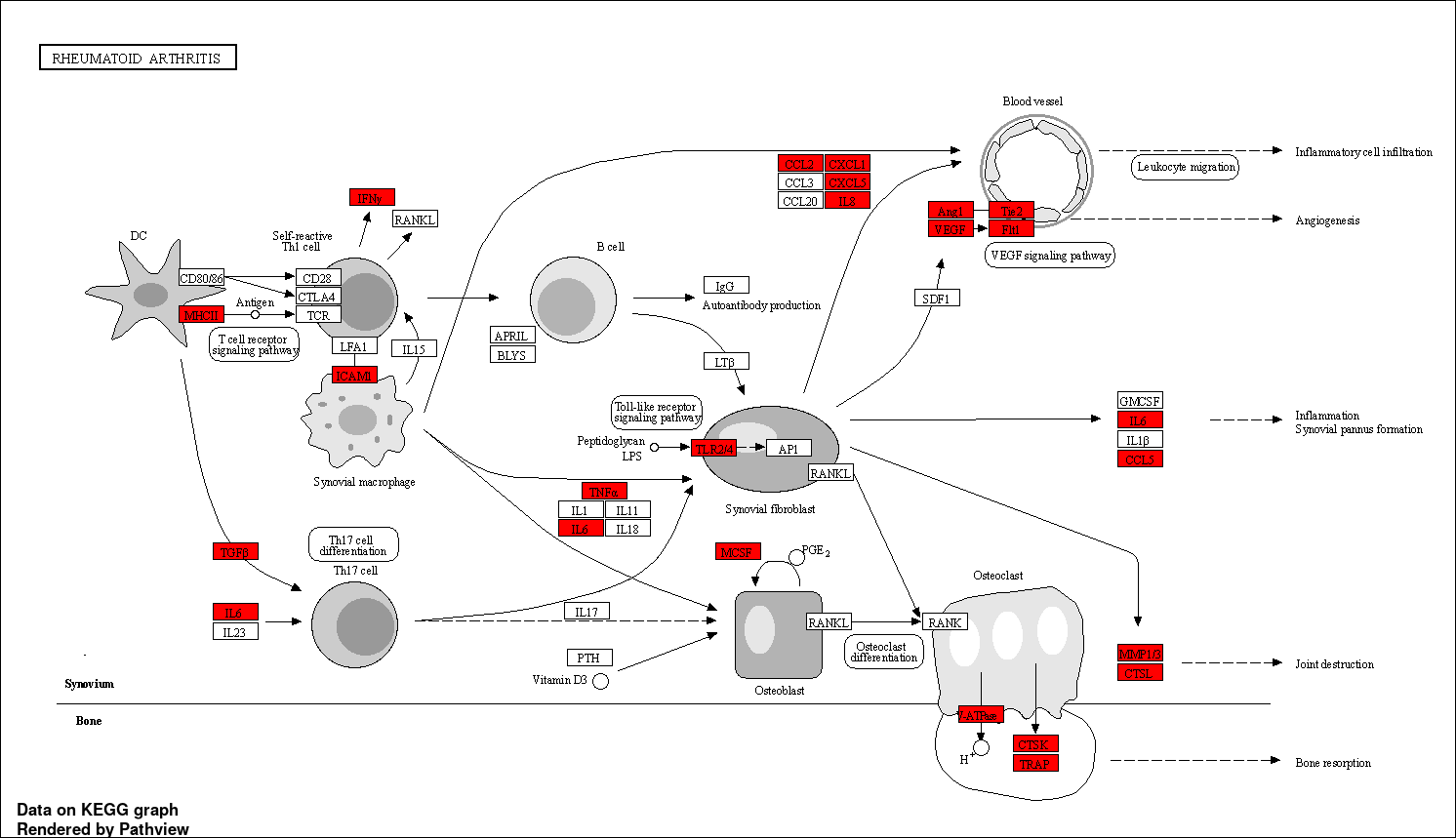


**Supplementary Figure 6. Therapeutical potential of PI-EVs and underlying mechanisms in Rheumatoid Arthritis.** Red nodes in the diagrams denote identified proteins in the corresponding EV-associated proteome. This KEGG diagram was created with ShinyGO 0.77 (http://bioinformatics.sdstate.edu/go/).

**Supplementary Tables**

**Supplementary Table 1. Human monoclonal antibody panel used for the characterization of MenSCs by flow cytometry.**

| **Antigen** | **Gene name** | **Fluorocrome** | **Company** | **Catalog number** | **Clone** |
| --- | --- | --- | --- | --- | --- |
| CD11b | CDK11B | FITC | Bio-Rad | MCA551F | ICRF44 |
| CD14 | CD14 | PE-CY5 | Bio-Rad | MCA2804C | 61D3 |
| CD29 | ITGB1 | FITC | Bio-Rad | MCA1949F | 4B7R |
| CD31 | PECAM1 | FITC | Bio-Rad | MCA1738F | WM59 |
| CD34 | CD34 | FITC | Bio-Rad | MCA547F | QBEND/10 |
| CD44 | CD44 | PE | BD | 550989 | 515 |
| CD45 | PTPRC | PE | BD | 555483 | HI30 |
| CD73 | NT5E | PE | BD | 550257 | AD2 |
| CD90 | THY1 | PE | Bio-Rad | MCA90PE | F15-42-1 |
| CD105 | ENG | PE | Bio-Rad | MCA1539PE | SN6 |
| CD117 | KIT | APC | Miltenyi Biotec | 130-091-733 | A3C6E2 |
| CD146 | MCAM | PercP | BD | 562135 | P1H12 |
| STRO1 | STRO1 | PE | INVITROGEN | MA5-28636 | STRO-1 |
| SUSD2 | SUSD2 | PE | Miltenyi Biotec | 130-117-794 | W5C5 |
| HLA-I | HLA-ABC | PE | BD | 555553 | G46-2.6 |
| HLA-II | HLAII DP DQ DR | FITC | Bio-Rad | MCA477F | WR18 |

**Supplementary Table 2. Antibody panel and immunoblotting conditions used for the characterization of basal MenSC-EVs analysed by SDS-PAGE.** Different proteins were detected under different SDS-PAGE settings including both non-reducing and reducing, denaturing conditions (obtained by absence or addition of DTT in the loading buffer, respectively). Blocking solutions, consisting on bovin serum albumin (BSA) or non-fat milk powder diluted in TBS-Tween 0.2% (TBS-T) buffer, as well as concentrations for the primary antibody solutions are indicated. Additional information regarding antibodies and band detection are also provided.

| **Antigen** | **Gene name** | **Observed molecular weight (MW)** | **SDS-PAGE conditions** | **Blocking, dilution conditions** | **Company** | **Catalog number** | **Clone** | **Host species** | **Signal detection method** |
| --- | --- | --- | --- | --- | --- | --- | --- | --- | --- |
| ALIX | *PDCD6IP* | 100 kDa | Reducing | BSA 5% in TBS-T, 1:500 | Santa Cruz Biotechnology | sc-53540 | 1A12 | Mouse | Chemiluminescence |
| CANX | *CANX* | 110/130 kDa | Non-reducing | Milk 2.5% in TBS-T, 1:1000 | Abcam | ab22595 | Polyclonal | Rabbit | Fluorescence |
| CD63 | *CD63* | 45-55 kDa | Non-reducing | Milk 2.5% in TBS-T, 1:1000 | Invitrogen | 10628D | Ts63 | Mouse | Chemiluminescence |
| CD81 | *CD81* | 25 kDa | Non-reducing | BSA 5% in TBS-T, 1:1000 | Abcam | ab79559 | M38 | Mouse | Chemiluminescence |
| FLOT1 | *FLOT1* | 50 kDa | Reducing | Milk 2.5% in TBS-T, 1:500 | BD Biosciences | 610821 | 18 | Mouse | Chemiluminescence |
| GAPDH | *GAPDH* | 37 kDa | Non-reducing | BSA 5% in TBS-T, 1:5000 | Sigma-Aldrich | G9545 | Polyclonal | Rabbit | Fluorescence |
| TSG101 | *TSG101* | 52 kDa | Reducing | Milk 2.5% in TBS-T, 1:1000 | Sigma-Aldrich (Prestige Antibodies) | HPA006161 | Polyclonal | Rabbit | Fluorescence |
